# Transcriptome of *Pectobacterium carotovorum* subsp. *carotovorum* PccS1 infected in calla plants in vivo highlights a spatiotemporal expression pattern of genes related to virulence, adaptation, and host response

**DOI:** 10.1111/mpp.12936

**Published:** 2020-04-08

**Authors:** Jiaqin Fan, Lin Ma, Chendi Zhao, Jingyuan Yan, Shu Che, Zhaowei Zhou, Huan Wang, Liuke Yang, Baishi Hu

**Affiliations:** ^1^ Laboratory of Bacteriology Department of Plant Pathology Nanjing Agricultural University Nanjing China

**Keywords:** adaptation, host response, infection in vivo, *Pectobacterium carotovorum*, transcriptome, virulence

## Abstract

Bacterial pathogens from the genus *Pectobacterium* cause soft rot in various plants, and result in important economic losses worldwide. We understand much about how these pathogens digest their hosts and protect themselves against plant defences, as well as some regulatory networks in these processes. However, the spatiotemporal expression of genome‐wide infection of *Pectobacterium* remains unclear, although researchers analysed this in some phytopathogens. In the present work, comparing the transcriptome profiles from cellular infection with growth in minimal and rich media, RNA‐Seq analyses revealed that the differentially expressed genes (log_2_‐fold ratio ≥ 1.0) in the cells of *Pectobacterium* *carotovorum* subsp. *carotovorum* PccS1 recovered at a series of time points after inoculation in the host in vivo covered approximately 50% of genes in the genome. Based on the dynamic expression changes in infection, the significantly differentially expressed genes (log_2_‐fold ratio ≥ 2.0) were classified into five types, and the main expression pattern of the genes for carbohydrate metabolism underlying the processes of infection was identified. The results are helpful to our understanding of the inducement of host plant and environmental adaption of *Pectobacterium*. In addition, our results demonstrate that maceration caused by PccS1 is due to the depression of callose deposition in the plant for resistance by the pathogenesis‐related genes and the superlytic ability of pectinolytic enzymes produced in PccS1, rather than the promotion of plant cell death elicited by the T3SS of bacteria as described in previous work.

## Graphical abstract

In *Pectobacterium* PccS1 infection, transcriptomic analysis using a two‐control gene expression experiment reveals more differentially expressed genes with different expression change types, which are related to virulence, adaptation, and host response.

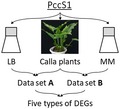

## 1 INTRODUCTION

In recent decades, advances in understanding how pathogenic bacteria respond to host plants and biotic or abiotic environmental factors have been proposed in many important phytopathogenic bacteria (Toth *et al.*, 2003; Charkowski *et al.*, 2012; Mansfield *et al.*, 2012; Ji *et al.*, 2016; Leonard *et al.*, 2017). Previous research revealed the characteristics of conservation and specificity in the gene expression of bacteria interacting with environmental factors. For example, in two phylogenetically distinct *Ralstonia solanacearum* strains recovered after inoculation in tomato plants, approximately 70% of the common orthologous genes expressed in a similar pattern (Jacobs *et al*., 2012), while profound differences were found in the global transcriptome of *Salmonella enterica* after in vitro growth in different media (Blair *et al.*, 2013). In our recent work, the proteomic profiles of *Pectobacterium carotovoum* revealed the influence of nutrients on gene expression, showing that some proteins were detected in the samples of cells recovered after being inoculated in the living host plant rather than in samples of the cells in medium supplemented with plant extracts (Wang *et al*., 2018b).

*P. carotovorum*, which causes rot, wilt, and blackleg in many crops and ornamental plants, results in important economic losses worldwide and is one of the top 10 plant pathogenic bacteria based on scientific/economic importance (Ma *et al.*, 2007; Mansfield *et al.*, 2012; Li *et al.*, 2018; Zhao *et al.*, 2018). Bacterial strains from the genera *Pectobacterium* and *Dickeya* are classified as soft‐rot *Enterobacteriaceae* (SRE). It is well known that SREs use plant cell wall‐degrading enzymes (PCWDEs) as the main pathogenic determinants to successfully infect host plants, and encode all six known protein secretion systems involved in attacking host plants and competing with environmental bacteria (Hugouvieux‐Cotte‐Pattat *et al.*, 1996; Bell *et al.*, 2004; Mole *et al.*, 2007; Charkowski *et al.*, 2012; Nykyri *et al.*, 2012; Joshi *et al*., 2016a). SREs secret PCWDEs mainly through a type II secretion system (T2SS) and digest their hosts more extensively than any other microbes. PCWDEs together with additional virulence factors, such as type III effector protein DspE and necrosis inducing protein Nip, are used to macerate plant tissue and promote plant cell death to provide nutrients for the multiplication and colonization of these necrotrophic pathogens in the course of infection (Kim *et al.*, 2011; Charkowski *et al.*, 2012; Haque *et al.*, 2017). Previous research suggested that elicitation of programmed cell death by a type III secretion system (T3SS) in plant leaves can promote virulence in *P. carotovorum* subsp. *carotovorum* (Kim *et al.*, 2011; Charkowski *et al.*, 2012). The T3SS deletion strains of SREs and the strain naturally lacking a T3SS can attack potato stems and tubers to a similar extent to those possessing a T3SS (Charkowski *et al.*, 2012). It is not known whether the inducement of plant cell death elicited by the T3SS is crucial to the virulence of SREs. Some regulators (RccR and HexR) controlling gene expression in response to carbon source availability in *Pseudomonas fluorescens* SBW25 and some genes (*pycA*, *aroBCD*, *eda*) encoding enzymes in the metabolic pathways of carbohydrates are crucial to the virulence of *Shigella flexneri*, *Listeria monocytogenes*, and *P. carotovorum* (Eisenreich *et al.*, 2010; Chavarria *et al.*, 2012; Campilongo *et al.*, 2017; Wang *et al*., 2018b), but we know little about these in *Pectobacterium*.

RNA‐Seq has been widely used in to help understand pathogen–plant interactions. Based on dynamic expression changes, RNA‐Seq approaches have identified genes that function in pathogen infection and adaption processes, such as pathogenicity, metabolism, signalling regulation, and response to complex environmental factors (Rio‐Alvarez *et al.*, 2012; Jiang *et al.*, 2015; Ah‐Fong *et al.*, 2017). A recent work with SREs revealed new functional insights into pathogenic determinants and interbacterial competition (Bellieny‐Rabelo *et al.*, 2019), but we know little about genome‐wide spatiotemporal expression during *Pectobacterium* infection. In the present work, we recovered cells of *P. carotovorum* subsp. *carotovorum* PccS1 at a series of time points after inoculation in the host plant, and compared the transcriptome profiles of the recovered cells with those from in vitro controls cultured in minimal and rich media. Over 2,000 significantly differentially expressed (log_2_‐fold ratio ≥ 2) genes (sDEGs) were identified and classified into five types of expression pattern. Some key genes that encode enzymes for acetyl‐CoA production from sucrose/glucose and pectin via pyruvate were significantly up‐regulated in PccS1 infection. While expression changes in genes for pathways of acid production were not widely detected, these results are helpful to our understanding of the bacterial response to environmental nutrients. Additionally, virulence in host plants and the hypersensitive response in nonhost plants were determined for mutants with one of the sDEGs deleted independently. The results demonstrate that the extensive host maceration by PccS1 is due to (a) the depression of callose deposition in plants by pathogenesis‐related genes or resistance being decreased by the host itself; and (b) the superlytic ability of pectinolytic enzymes in *Pectobacterium* PccS1.

## 2 RESULTS AND DISCUSSION

### 2.1 Symptoms and bacterial population in the course of *Pectobacterium* PccS1 infection in planta

To visualize transcriptome analyses at different time points after PccS1 inoculation, calla lily plant symptoms were continuously observed from inoculation of PccS1 onto the petioles of plants until the petioles had nearly fallen (Figure S1), and the quantity of PccS1 cells in planta was measured as previously described (Jiang *et al.*, 2017). The results revealed that PccS1 multiplied in planta slowly in the early infection stage (before 4 hr after inoculation, HAI). The population was nearly 5 × 10^6^ cfu at 12 HAI, and then increased quickly (Figure S2), similar to previous results that demonstrated that the threshold of bacterial population should be 10^6^–10^7^ cfu to effectively elicit expression of the genes participating in a variety of cell density‐dependent physiological processes (Toth *et al.*, 2003; Ng and Bassler, 2009; Hawver *et al*., 2016).

### 2.2 DEGs in PccS1 recovered from inoculated plants

To examine the spatiotemporal expression of genes in *Pectobacterium* PccS1 infection in calla lily plants, a whole‐transcriptome data set was generated, including samples of PccS1 recovered 4, 8, 12, and 16 HAI in plants or grown in media (Luria Bertani [LB] or minimal medium [MM]). Our results showed a good overall quality of clean reads with Q20 > 98% obtained in each sample, and over 97% genes in the reference genome were uniquely mapped. Subsequent analyses showed that 48.72% and 57.50% of genes of the cells recovered were differentially expressed (log_2_‐fold changed ≥ 1.0, false discovery rate [FDR] < 0.05) at the early stage of infection (4 HAI) versus that of the cultures in LB and MM, respectively (Table S1.1). Recent studies on transcriptome sequencing of closely related SRE strains detected 13.5% of *Dickeya dadantii* genes differentially expressed during the early stages of interaction with *Arabidopsis thaliana* between the *pecS* mutant and wild type (Pédron *et al*., 2018), and identified 43.5% of *P. carotovorum* subsp. *brasiliense* 1692 genes under infection‐induced regulation by comparing the samples of potato tuber infection cells with the in vitro control in LB medium (Bellieny‐Rabelo *et al.*, 2019).

More DEGs were identified from the data set when PccS1 in MM was used as the control than when PccS1 in LB was used as the control. Between the data sets with different controls, the quantity of differences of DEGs in PccS1 recovered at 4, 8, 12, and 16 HAI were 8.78%, 15.98%, 14.37%, and 11.81% of the total annotated gene number, respectively (Table S1.1). Our results agree with previous research that showed that the growth medium of bacteria is an important consideration during experimental design for any experimental protocol (Blair *et al.*, 2013). For example, a total of 621 genes were found to be differentially expressed when *S. enterica* SL1344 was grown in MOPS MM compared to growth in LB (Blair *et al.*, 2013).

**FIGURE 1:**
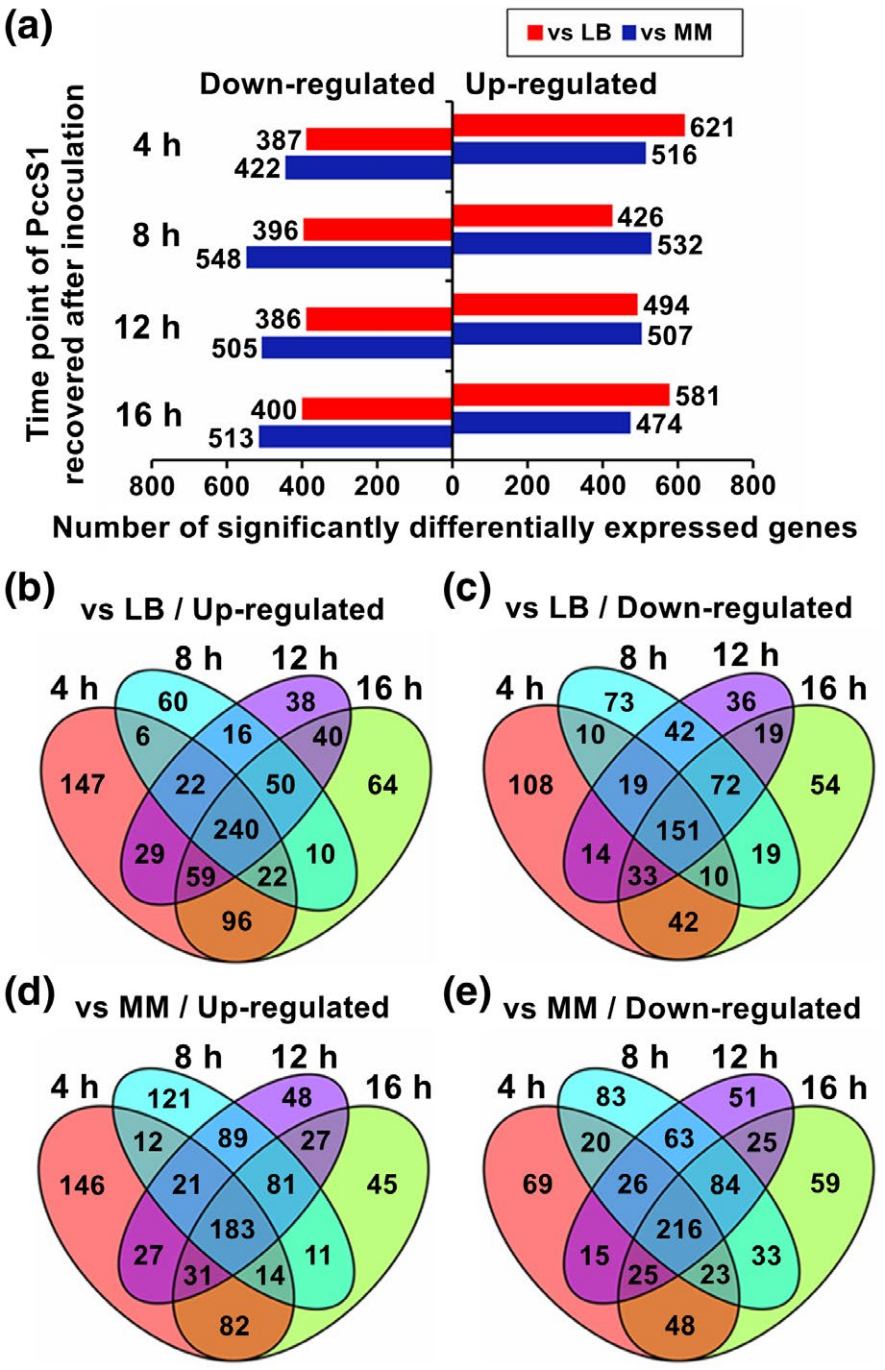
The number of significantly differentially expressed genes (ratio of log_2_‐fold ≥ 2) (sDEGs) in *Pcetobacterium carotovorum* subsp. *carotovorum* PccS1 recovered at four different time points after inoculation in *Zantedeschia odorata* plants compared with cells in Luria Bertani medium (LB) and minimal medium (MM). (a) Total number of sDEGs in PccS1 recovered compared with the LB and MM controls. (b)–(e) Venn diagrams showing number of sDEGs up‐regulated and down‐regulated in PccS1 recovered compared with the controls

Based on the stringency of the ratio of log_2_‐fold ≥ 2.0 and a threshold FDR of 0.05 or less, the sDEGs in PccS1 infection in planta at different time points were obtained (Tables S1.2 and S1.3), and the numbers of sDEGs in each sample were calculated (Figure 1a). There were 391 and 399 sDEGs present at all four time points compared with the in vitro controls in LB and MM, respectively (Figure 1b–e). Most of the genes in the T3SS and T6SS clusters, and some genes for pectate lyase and protease (Tables S1.2 and S1.3), were included in the consistently up‐regulated sDEGs in PccS1 infection. Some of these genes have been demonstrated to be important for the virulence of SRE (Koo *et al.*, 2012; Bondage *et al.*, 2016; Pédron *et al*., 2018). In some closely related SRE, genes in the T6SS cluster and for pectate lyase were also found to be up‐regulated during infection (Bellieny‐Rabelo *et al.*, 2019).

### 2.3 Validation of DEGs by quantitative reverse transcription PCR

**FIGURE 2:**
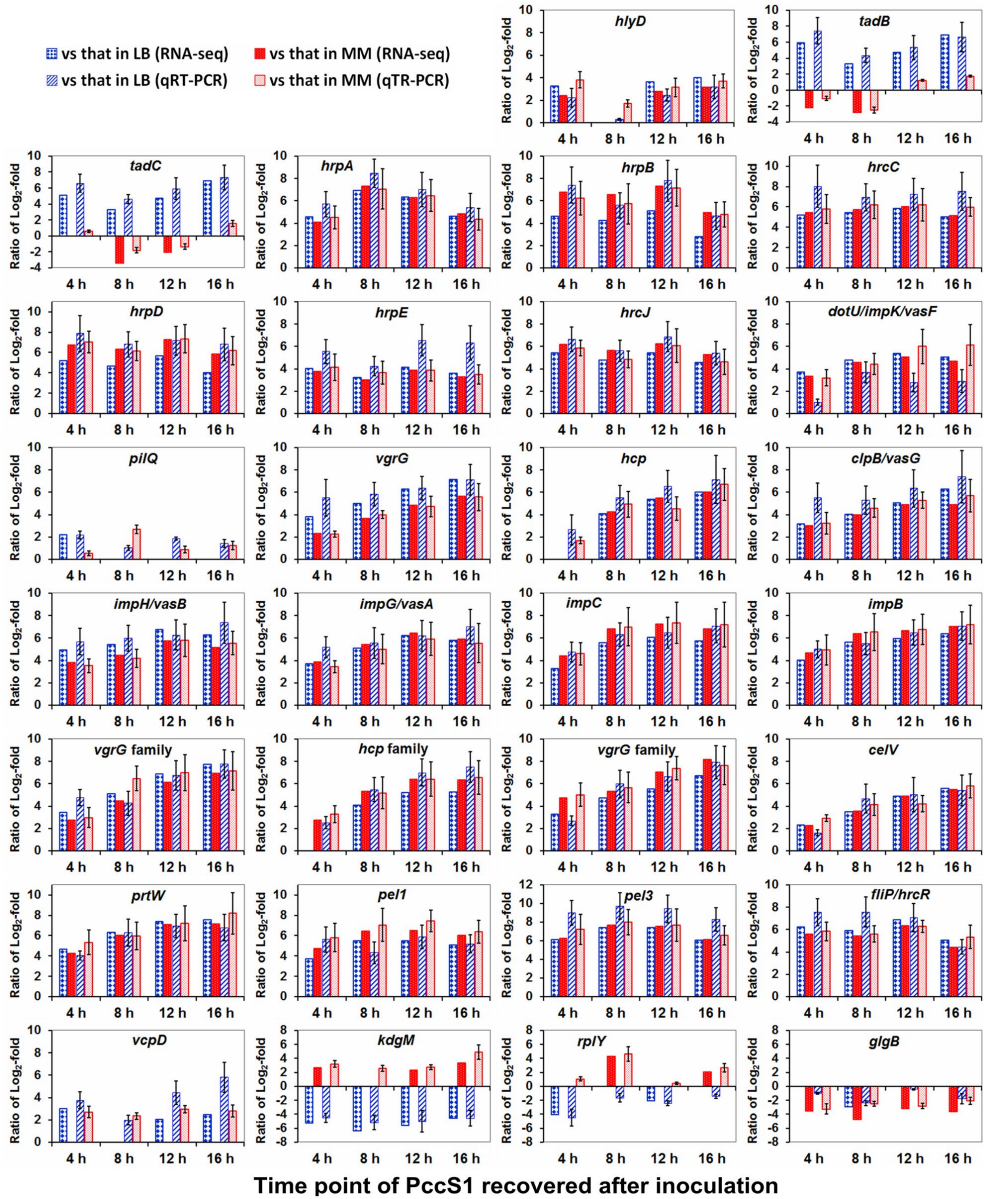
Ratios of the expression levels of 30 genes between *Pectobacterium* PccS1 recovered and the cultures in Luria Bertani (LB) and minimal medium (MM) using quantitative reverse transcription PCR (RT‐qPCR) and RNA‐Seq analyses. Bars without standard errors indicate ratios of transcript abundance changes between PccS1 recovered and the controls according to RNA‐Seq data (log_2_‐fold ratio ≥ 2 for at least one time point in PccS1 recovered versus the controls). Bars with standard errors represent the ratios of gene expression between PccS1 recovered and the controls determined by RT‐qPCR

To verify the results of DEGs identified from Illumina sequencing data, a total of 30 genes were selected randomly from the sDEGs for quantitative reverse transcription PCR (RT‐qPCR) analysis, as previously described (Allie *et al.*, 2014; Wang *et al.*, 2015). The gene expression trends of the selected genes in each sample were analysed using RT‐qPCR and the results were mainly consistent (Figure 2), although variations in the exact fold changes were observed between the results of RT‐qPCR and RNA‐Seq, possibly due to differences in the sensitivity and specificity between these two approaches. The data demonstrate that the RNA‐Seq data accurately reflect the response of *Pectobacterium* PccS1 to the establishment of infection in *Zantedeschia odorata* plants, as seen in previous work (Allie *et al.*, 2014; Wang *et al.*, 2015; Skorupa *et al.*, 2016). Meanwhile, to evaluate gene expression, the expression levels of some housekeeping genes, such as *recA*, *gyrB*, *infB*, and *rpob*, were compared in the samples; the results showed no significant expression changes in each sample of either PccS1 in planta or the in vitro controls, indicating that the RNA‐Seq data are comparable with previous work (Shen *et al.*, 2016).

### 2.4 Functional characterization of the sDEGs in PccS1 recovered after inoculation

**FIGURE 3:**
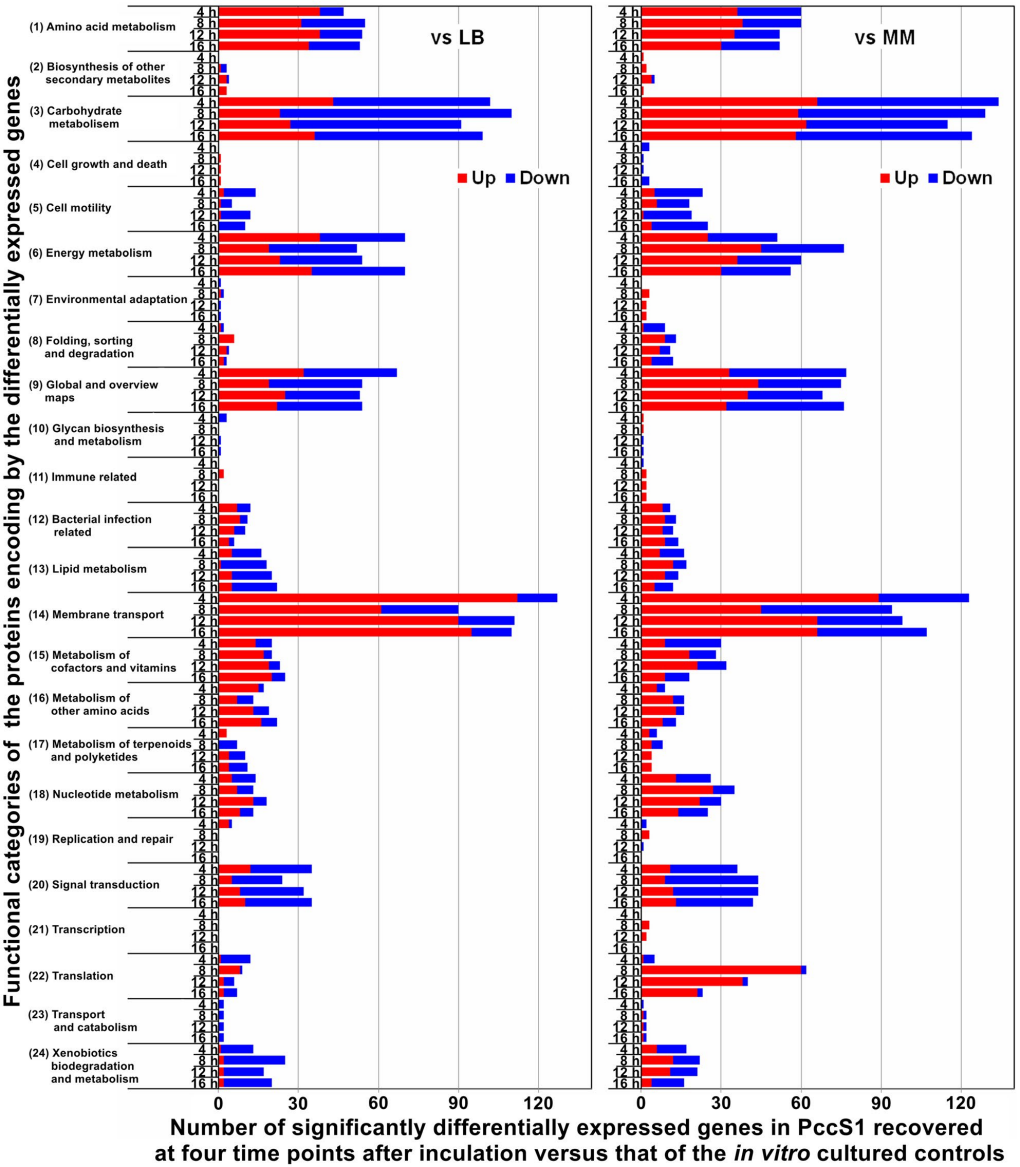
Distribution of significantly differentially expressed genes among the functional categories of the KEGG pathway annotation in *Pectobacterium* PccS1 recovered compared with the controls from the cultures in Luria Bertani (LB) and minimal medium (MM)

To understand the biological significance of the sDEGs in the course of PccS1 infection, we characterized the functions of the proteins encoded by the sDEGs at four time points by mapping the genes to the Kyoto Encyclopedia of Genes and Genomes (KEGG) database (http://www.genome.jp/keeg/) (Figure 3). The results reveal that the functional categories of the proteins encoded by the sDEGs were mainly involved in three KEGG pathways (membrane transport, carbohydrate metabolism, and energy metabolism) in PccS1 recovered at the four time points, as compared with the in vitro controls cultured in either LB or MM (Figure 3). The expression of the sDEGs for these categories of protein showed a consistent pattern of regulation in the course of infection: most of the sDEGs encoding proteins in membrane transport were up‐regulated, those for carbohydrate metabolism were down‐regulated, and approximately half of those for energy metabolism were up‐regulated and the other half were down‐regulated (Figure 3). In some protein categories, more sDEGs were observed in the data set of PccS1 infection compared with growth in MM than for PccS1 infection compared with growth in LB, such as those belonging to carbohydrate metabolism, translation, signal transduction, cell motility, and nucleotide and amino acid metabolism (Figure 3). Previous research showed that genes in amino acid biosynthetic pathways were up‐regulated in *S. enterica* and *Escherichia coli* grown in MM as compared with the bacteria grown in LB (Tao *et al.*, 1999; Blair *et al.*, 2013). The proteins encoded by the sDEGs with increased expression in PccS1 infection are probably involved in bacterial–plant interactions and bacterial multiplication, representing genes involved in the successful colonization and adaption of PccS1 to different nutrient levels.

### 2.5 Classification of the *Pectobacterium* PccS1 sDEGs by the expression pattern in the processes of infection

There were 2,281 sDEGs in the trancriptome pools from PccS1 recovered at the four time points after inoculation versus the reference genes from the cells in either LB or MM. The 2,281 sDEGs were classified into five types to visualize the different types of gene expression change under different nutrient levels (Table 1): (a) plant‐induced genes were differentially expressed (either up‐regulated or down‐regulated) in the bacteria recovered from the plants compared to both the control media; (b) basal‐nutrient‐induced genes were differentially expressed in the bacteria recovered from plants compared with the MM control, while they were similarly expressed to the LB control; (c) rich‐nutrient‐induced genes were differentially expressed in the bacteria recovered from plants compared to the LB control, but were similar to the MM control; (d) nutrient‐level susceptible genes in the recovered bacteria were not only differentially expressed compared with both the control media, but also exhibited different regulation (up and down) in comparison with the different media (MM and LB); and (e) special genes were expressed in the recovered bacteria with different change tendencies at different infection stages compared with the two controls. *PccS1_00151* and *PccS1_00152* were classified as special genes (Tables 2, S1.2, and S1.3) as their expression levels were linked to the infection stage. Genes in each category were further identified as a subtype of positive or negative by the tendencies of expression change as compared with the controls, except the two special genes (Table 1). Thirty genes with a log_2_‐fold ratio larger than 10 for at least one recovered time point versus the reference genes are listed in Table 2. None of the rich‐nutrient negatively induced genes had a log_2_‐fold ratio of expression change larger than 10 (Table S1.2 and S1.3).

**TABLE 1:** Types of significantly differentially expressed genes showing different expression patterns in *Pectobacterium* PccS1 recovered from *Zantedeschia odorata* at different times after inoculation when compared with the cells in Luria Bertani medium (LB) or minimal medium (MM)

Type	Subtype	Expression pattern		Quantity
		vs that in LB	vs that in MM	
Plant‐induced gene	Positive	Up	Up	518
	Negative	Down	Down	357
Basal‐nutrient‐induced gene	Positive	Similar	Down	349
	Negative	Similar	Up	333
Rich‐nutrient‐induced gene	Positive	Down	Similar	258
	Negative	Up	Similar	245
Nutrient‐level susceptible gene	Positive	Down	Up	85
	Negative	Up	Down	134
Special gene	‐	Up initially, then down	Up initially, then same as	2
Total				2,281

**TABLE 2:** The significantly differentially expressed genes in *Pectobacterium* PccS1 recovered versus that of the controls of the cells cultured in Luria Bertani medium (LB) and minimal medium (MM) with the log_2_‐fold ratio larger than 10 at least at one recovered time point after inoculation are classified into five types based on the tendency of expression changed under different growth conditions

Type	Subtype	Gene name	Gene ID	Ratio of log_2_‐fold (vs that in LB)				Ratio of log_2_‐fold (vs that in MM)			
				4 hr	8 hr	12 hr	16 hr	4 hr	8 hr	12 hr	16 hr
Plant‐induced gene	Positive	*nrtA*	*PccS1_04055*	9.73	8.39	**10.18**	8.68	8.02	6.80	8.51	6.94
	Negative	*rsmA*	*PccS1_00073*	**−10.39**	—	−3.93	—	−8.35	—	−3.14	—
			*PccS1_00094*	−3.25	−2.36	−5.35	**−10.82**	−2.90	—	−4.94	−9.16
			*PccS1_00098*	−3.25	−2.36	−5.35	**−10.82**	−2.90	—	−4.95	−9.16
		*gloA*	*PccS1_00200*	−2.21	−4.94	**−11.43**	−2.96	−2.95	−5.57	−**10.97**	−3.73
			*PccS1_00338*	−2.34	−5.26	−6.43	**−11.89**	—	−3.90	−2.70	−9.34
			*PccS1_01232*	**−11.31**	−4.67	−5.86	—	**−10.52**	−5.06	−6.28	−2.71
			*PccS1_01413*	—	—	—	**−10.15**	—	−2.03	—	−9.23
			*PccS1_01418*	—	−2.77	−3.01	−**12.69**	—	—	—	−**10.39**
			*PccS1_01956*	−3.59	−6.59	−6.07	−**15.31**	—	−2.76	−2.31	−**10.28**
			*PccS1_01959*	—	−3.46	−2.26	−**12.09**	−2.30	−4.77	−3.64	−**12.19**
			*PccS1_02071*	—	−3.44	−**10.66**	—	—	−2.39	−8.51	—
		*rpmE*	*PccS1_02329*	—	—	—	−8.76	−5.57	−5.42	−5.66	−**10.85**
			*PccS1_02355*	—	—	−2.88	−**10.32**	—	—	—	−8.27
			*PccS1_02573*	−2.25	−3.92	−4.95	−**11.98**	—	−2.95	−4.05	−9.81
			*PccS1_02574*	—	−2.43	−2.51	−**11.41**	—	−2.34	−2.50	−**10.08**
			*PccS1_02645*		−2.37	−3.15	−**10.18**	—	−2.39	−3.25	−9.01
			*PccS1_02981*	−2.42	−4.31	−3.86	−**11.30**	—	−3.13	−2.75	−8.92
			*PccS1_03434*	—	−3.57	−3.94	−**10.97**	—	−4.47	−4.91	−**10.67**
		*narP*	*PccS1_03762*	−**11.16**	−2.39	−4.12	−2.76	−**11.20**	−3.62	−5.42	−4.13
			*PccS1_04007*	−**11.09**	—	−4.05	−3.26	−9.68		−3.90	−3.19
Basal‐nutrient‐induced gene	Positive		*PccS1_01325*	—	—	—	—	−6.26	−6.73	−6.35	−**12.52**
	Negative	*pfk*	*PccS1_03311*	—	—	—	—	**10.07**	7.78	8.04	8.27
Rich‐nutrient‐induced gene	Positive		*PccS1_00101*	−3.79	−3.72	−4.90	−**11.35**	—	—	—	—
			*PccS1_01957*	—	−**10.05**	−4.26	−9.73	—	—	—	—
			*PccS1_02999*	—	−3.77	−2.44	−**11.05**	—	—	—	—
			*PccS1_03529*		−2.35	−4.87	−**10.33**	—	—	—	—
	Negative			**>1.00**				—	—	—	—
Nutrient‐level susceptible gene	Positive		*PccS1_01834*	−**12.11**	—	−2.34	−2.70	—	4.76	2.86	—
	Negative	*Flp/fap*	*PccS1_01974*	—	8.22	**10.27**	**10.13**	−3.95	−4.50	−2.29	−2.42
Special gene			*PccS1_00152*	2.15	—	—	−**10.08**	3.13	—	—	—

In previous work, we identified “infection‐induced regulation genes” in the transcriptome data of bacterial infection compared with the in vitro control in LB medium (Pédron *et al*., 2018; Bellieny‐Rabelo *et al.*, 2019). We propose the classification of the sDEGs based on the expression changes in PccS1 in planta versus the two controls. If the sample in MM had not been used as a control, the sDEGs that were classified as “basal‐nutrient‐induced genes” would not have been obtained, highlighting the importance of media choice. For example, *pfk* (Table 2) did not present as one of the DEGs identified in the wild‐type *D. dadantii* interaction with *A. thaliana* versus that of the cultures in LB only (Pédron *et al*., 2018). In the experiments on PccS1, 682 sDEGs were identified that exhibited differential expression relative to MM (Table 1). If the samples in LB had been used as the sole control, “plant‐induced,” “rich‐nutrient‐induced”, and “nutrient‐level susceptible” genes would have been identified as “infection‐induced regulation genes,” although they are not the same based on the expression changes in PccS1 infection versus in MM. This example represents an advantage of a gene expression experiment based on the comparison of RNA‐Seq data with two controls. By using a two‐control gene expression experiment, we obtained DEGs including not only infection‐induced genes but also nutrient‐adaption genes, although the classification of genes with different expression patterns in PccS1 infection in different controls requires further support from studies on other pathogenic bacteria.

In our recent work, we obtained a transposon library of PccS1 with kanamycin resistance dependent on the host plant in the mimic test (MM supplemented with 0.3% *Zantedeschia elliotiana* extract) (Jiang *et al.*, 2017), a host plant that is closely related to *Z. odorata* used in this work. Only one transposon insertion mutant showed attenuated virulence in the host plants; the gene at the insertion site of the mutant was characterized as *rplY* (Jiang *et al.*, 2017), which exhibited a similar expression pattern in planta and in the mimic test. However, in this study, the expression pattern of *rplY* in PccS1 in planta should be classified as “nutrient‐level susceptible” (Figure 2 and Table 1). To some extent, this reveals the effects of host nutrients on bacterial adaptation. Similarly, genome comparison has revealed a predicted benzoic acid/salicylic acid carboxyl methyltransferase that relates to life in planta and other specific environmental conditions in *Pectobacterium wasabiae* SCC3193 (Nykyri *et al.*, 2012). Three *P. wasabiae* strains, including SCC3193, collected from potato, constituted a separate clade from the original *P. wasabiae* strain from Japanese horseradish using multilocus sequence analysis. The separate clade was validated by DNA–DNA hybridization and genome average nucleotide identity, and the three potato strains were transferred to a proposed new species called *Pectobacterium parmentieri* (Khayi *et al.*, 2016). Our data confirm that global transcriptomes from bacterial cells grown in different conditions are profoundly different, and that choice of medium should be considered carefully during experimental design (Blair *et al.*, 2013). Gene characterization and further study of their functions based on nutrient utilization will enhance our understanding of bacterial adaptation to the host (Anderson and Kendall, 2017).

### 2.6 Expression of genes related to the main virulence determinants during *Pectobacterium* PccS1 infection

The PCWDEs, including pectinase, cellulase, and protease, have been demonstrated to be the main virulence determinants in SRE that cause extensive plant tissue maceration (Toth *et al.*, 2006; Wang *et al*., 2018b). The *Pectobacterium* PccS1 genome contains 27 pectinase‐encoding genes (Table 3), including 13 genes for pectate lyase, 7 for polygalacturonase, and 7 for pectin‐methyl‐ and pectin‐acetyl‐esterase. Among the genes for pectate lyase, six genes (three *pel1* genes, two *pel3* genes, and one *pelL* gene) were up‐regulated in infection, belonging to the positively plant‐induced gene type. These results emphasize the role of pectate lyase (PL) in PccS1 infection, which is similar to previous work that showed that all nine PLs (*pelABCDEILNZ*) in *D. dadantii* (Pédron *et al*., 2018) and seven out eight PLs in *P. carotovorum* subsp. *brasiliense* 1692 (Bellieny‐Rabelo *et al.*, 2019) were positively induced during infection of host plants. In contrast, *pelB*, a gene encoding periplasmic pectate lyase, was down‐regulated compared with the LB control, but up‐regulated in comparison with the MM control, and classified as a nutrient‐level susceptible gene. Expression of most of the genes in the other two kinds of pectinases (polygalacturonase and pectin‐esterases) was not significantly changed in PccS1 infection compared to the references, except that four genes for polygalacturonases (*pehX* and three *peh* genes) were differentially expressed with a ratio of log_2_‐fold of approximately 2–3 in the samples of PccS1 recovered at some time points (Table 3).

**TABLE 3:** Log_2_‐fold ratios of the genes encoding exo‐enzymes in *Pectobacterium* PccS1 recovered from *Zantedeschia odorata* at different times after inoculation compared with that of the cells grown in the Luria Bertani medium (LB) and minimal medium (MM)

Function (annotated with multi‐databases)	Gene name	Gene ID	Ratio of log_2_‐fold (vs that in LB)				Ratio of log_2_‐fold (vs that in MM)			
			4 hr	8 hr	12 hr	16 hr	4 hr	8 hr	12 hr	16 hr
Dienelactone hydrolase	*pel10*	*PccS1_00443*	—	—	—	—	2.28	—	—	2.11
Pectate lyase/Amb allergen	*pel1*	*PccS1_00814*	3.74	5.35	5.49	5.09	4.73	6.45	6.51	6.05
Pectate lyase	*pel2*	*PccS1_00815*	—	—	—	—	—	—	2.03	2.40
Pectate lyase/Amb allergen	*Pel3*	*PccS1_00816*	—	2.14	2.95	3.12	2.88	3.35	4.09	4.18
Pectate lyase	*pel1*	*PccS1_00817*	2.53	2.82	4.08	4.35	3.50	3.90	5.08	5.28
Pectate disaccharide lyase	*pel9*	*PccS1_01252*	—	—	—	—	2.61	—	—	2.53
Pectate lyase	*pel3*	*PccS1_02251*	2.10	3.79	5.03	4.89	2.43	5.23	5.29	5.18
Pectate lyase/Amb allergen	*pel1*	*PccS1_02679*	—	3.39	3.46	3.64	—	4.90	4.90	5.00
Pectate lyase	*pelL*	*PccS1‐03128*	—	3.42	4.56	4.15	—	3.58	4.64	4.16
Pectate disaccharide lyase	*pelW*	*PccS1_03263*	—	—	—	—	—	—	—	—
Periplasmic pectate lyase	*PelB*	*PccS1_03525*	−4.14	−3.23	−3.90	−2.53	—	—	—	2.34
Pectate lyase	*pel3*	*PccS1_03537*	6.15	7.42	7.40	6.05	6.29	7.67	7.57	6.15
Pectate lyase/Amb allergen	*pel1*	*PccS1_04181*	—	—	—	—	2.33	—	—	—
6‐phosphogluconolactonase	*pgl*	*PccS1_00206*	—	—	—	—	—	—	—	—
Glycoside hydrolase	*peh*	*PccS1_00253*	2.06	—	—	—	2.10	—	—	—
Glycoside hydrolase	*peh*	*PccS1_02252*	—	—	—	2.93	−2.87	—	—	—
Exo‐poly‐α‐D‐galacturonosidase	*peh*	*PccS1_02352*	—	—	—	—	2.30	2.03	2.29	3.02
Threonine‐tRNA ligase	*thrS*	*PccS1_03243*	—	—	—	—	—	—	—	—
Glycoside hydrolase	*peh*	*PccS1_03482*	—	—	—	—	—	—	—	—
Endopolygalacturonase	*pehX*	*PccS1_04180*	−2.49	—	−2.54	−2.46	−2.02	—	−2.05	−2.03
Isomerase	*kduI*	*PccS1_00873*	—	—	—	—	—	—	—	—
Acyl‐CoA thioesterase	*pemB*	*PccS1_01371*	—	—	—	—	—	—	—	—
Oligogalacturonide lyase	*ogl*	*PccS1_03239*	—	—	—	—	—	—	—	—
Pectin acetylesterase	*pea*	*PccS1_03257*	—	—	—	—	—	—	—	—
Isomerase	*kduI*	*PccS1_03265*	—	—	—	—	—	—	—	—
Pectin acetylesterase	*peaA*	*PccS1_04335*	—	—	—	—	—	—	—	—
Pectin methylesterase A	*pemA*	*PccS1_04336*	—	—	—	—	—	—	—	—
Cellulase (precursor)		*PccS1_01134*	—	−2.61	—	—	—	−3.22	−2.28	—
β‐(1,4)‐glucan glucanohydrolase	*celB*	*PccS1_02846*	−2.14	−2.28	—	—	2.13	2.10	3.01	3.11
Putative cellulase	*cel*	*PccS1_03445*	—	4.40	5.04	4.91	4.03	6.68	7.24	7.04
Cellulose binding/Endoglucanase	*celV*	*PccS1_03690*	2.30	3.50	4.90	5.59	2.26	3.57	4.89	5.51
Serine 3‐dehydrogenase	*prtC*	*PccS1_02900*	4.65	6.31	7.42	7.60	4.27	6.04	7.07	7.18
Metalloprotease inhibitor	*inh*	*PccS1_02901*	3.84	3.32	5.17	6.28	—	—	2.58	3.63
ATP‐type protease	*prtD*	*PccS1_02902*	3.93	3.13	5.21	5.82	2.43	—	3.74	4.28
HlyD family membrane fusion protein	*prtE*	*PccS1_02903*	2.94	—	3.12	3.89	—	—	—	2.18
TolC family outer membrane protein	*prtF*	*PccS1_02904*	2.55	—	—	2.70	—	—	—	—

Three cellulose genes (*cel, celV*, and a gene for cellulose precursor) and five protease genes (*prtC, D, E, F* and *inh*) are positively plant‐induced genes, and *celB* , like *rplY*, is a nutrient‐level susceptible gene (Table 3). This indicates the importance of protease genes in the infection of SRE. Similarly, *prtA, B, C, D, E, F, G* and *inh* were up‐regulated in the *D. dadantii* interaction with *A. thaliana* at 6 and 24 HAI as compared with the in vitro control grown in LB (Pédron *et al*., 2018).

In our recent work, we demonstrated that genes crucial to virulence, such as *rplY*, *eda*, *hfq*, and *flgK*, significantly affected the activities of PCWDEs at both the transcriptional and translational levels in PccS1 (Yang *et al.*, 2012; Jiang *et al.*, 2017; Wang *et al*., 2018a, 2018b). Our transcriptome data revealed that the longer the time of PccS1 infiltration in the host, the higher the expression level of most of the genes for PCWDEs during the course of infection (Table 3). This demonstrates that the RNA‐Seq data accurately reflect the response of *Pectobacterium* PccS1 to the establishment of infection in *Z. odorata*. It also shows a relationship between substrate (pectin) metabolism and the regulation of PCWDE expression, to some extent, which represents the adaption of PccS1 gene expression to environmental nutrient status.

### 2.7 Expression of the genes related to the regulators of virulence

The regulatory networks of the main virulence determinants in SREs have been examined, focusing on the genes related to acyl‐homoserine lactone (AHL) (such as *carI* and *expR*) and on those for regulation of pectin catabolism (*kdgR*, *rsmA*, *rsmB*) (Charkowski *et al.*, 2012). Pathogenic bacteria possess specific transcriptional regulators to sense sophisticated changes (osmotic, acidic, and anaerobic stress, redox potential, etc.) in planta and in natural environments, which in turn affect interaction with plants or microbes nearby (Babujee *et al.*, 2012; Reverchon and Nasser, 2013; Broberg *et al.*, 2014; George *et al*., 2018). Based on these previous studies, we identified 41 genes encoding regulatory proteins of virulence in the PccS1 genome, and compared their expression levels in planta to the references (Table 4).

**TABLE 4:** Log_2_‐fold ratios of the genes related to regulatory networks in *Pectobacterium* PccS1 recovered from *Zantedeschia odorata* at different times after inoculation compared with that of the cells grown in Luria Bertani medium (LB) and minimal medium (MM)

Function (annotated with multiple databases)	Gene name	Gene ID	Ratio of log_2_‐fold (vs that in LB)				Ratio of log_2_‐fold (vs that in MM)			
			4 hr	8 hr	12 hr	16 hr	4 hr	8 hr	12 hr	16 hr
RNA small subunit methyltransferase A	*rsmA*	*PccS1_00073*	−**10.38**	—	−3.92	—	−**8.35**	—	−3.14	—
RNA small subunit methyltransferase A	*rsmA*	*PccS1_00521*	—	—	—	—	—	—	—	—
rRNA (cytosine‐C(5)‐)‐methyltransferase	*rsmB*	*PccS1_00733*	—	—	—	—	—	—	—	—
Transcription anti‐termination protein	*nusB/rsmB*	*PccS1_02289*	—	—	—	—	—	—	—	—
rRNA (guanine‐N(2)‐)‐methyltransferase	*rsmC*	*PccS1_01829*	—	—	—	—	—	—	—	—
rRNA (guanine‐N(2)‐)‐methyltransferase	*rsmC*	*PccS1_01902*	—	—	—	—	—	2.64	2.18	—
RNA polymerase, σ subunit	*rpoS*	*PccS1_00230*	−2.53	−2.19	−2.32	−3.03	—	—	—	—
AHL synthase	*carI*	*PccS1_01369*	—	—	—	—	—	—	−2.33	−2.15
Transcriptional activator protein, LuxR	*expR*	*PccS1_01370*	—	—	—	−2.24	—	—	—	—
Regulation of transcription, IclR family	*kdgR*	*PccS1_03240*	—	—	—	—	—	—	—	—
Nitrate/nitrite sensor protein	*narX*	*PccS1_03626*	—	—	—	—	—	—	—	—
Two‐component transcriptional regulator	*narL*	*PccS1_03627*	—	—	—	—	−3.64	−2.79	−3.48	−3.76
Nitrogen regulation protein, NR(I)	*ntrC*	*PccS1_01293*	2.35	—	2.38	—	—	−3.09	—	−2.52
Nitrogen regulation protein NR(II)	*ntrB*	*PccS1‐01294*	2.75	—	2.71	2.18	—	−3.31	—	−2.23
Hydroperoxide‐inducible gene activator	*oxyR*	*PccS1_03709*	—	—	—	—	—	—	—	—
Hydroperoxide resistance transcriptional regulator	*ohrR*	*PccS1_04238*	—	—	—	—	—	—	—	—
Response protein/histidine kinase	*phoQ*	*PccS1_03221*	—	—	—	—	—	—	−2.08	—
Two‐component transcriptional regulator	*phoP*	*PccS1_03222*	−3.38	—	—	—	−2.57	—	—	—
Transcriptional repressor, MarR family	*marR/mprA*	*PccS1_00210*	—	—	—	—	—	—	—	—
Transcriptional regulator, MarR family	*marR*	*PccS1_00313*	—	—	—	—	—	—	—	—
Transcriptional regulator, MarR family	*marR*	*PccS1_02127*	—	—	—	—	—	—	—	—
Transcriptional regulator, MarR‐type HTH domain		*PccS1_02470*	—	—	—	—	—	—	—	—
Transcriptional regulator, MarR family	*marR*	*PccS1_03609*	—	—	—	—	—	—	—	—
Transcriptional regulator, MarR family	*marR/sylA*	*PccS1_03735*	—	—	—	—	−3.10	−2.31	−2.37	−2.28
Transcriptional regulator, MarR family	*marR*	*PccS1‐03755*	—	—	—	—	—	—	—	—
Transcriptional regulator, MarR family	*marR*	*PccS1_04238*	—	—	—	—	—	—	—	—
Anaerobic regulatory, FNR/CRP family	*fnr*	*PccS1_03460*	—	—	—	—	—	—	—	—
Anaerobic regulatory protein	*Crp/fnf*	*PccS1_03828*	2.52	—	2.30	—	—	−2.53	—	—
Anaerobic nitric oxide reductase transcriptional regulator	*norR*	*PccS1_02070*	—	—	—	2.14	2.01	2.13	—	2.79
Aerobic respiration control protein	*arcA*	*PccS1_00560*	−2.01	—	—	—	—	—	—	—
Aerobic respiration control sensor	*arcB*	*PccS1_01588*	—	—	—	—	—	—	—	—
DNA‐binding protein	*fis*	*PccS1_01523*	—	3.22	—	—	2.66	4.83	2.49	2.10
σ‐54 transcriptional regulator	*fis*	*PccS1_03556*	**5.10**	2.96	4.31	4.29	3.61	—	2.86	2.77
σ‐54 transcriptional activator	*fis/pspF*	*PccS1_03689*	—	—	—	—	—	—	—	—
DNA mismatch endonuclease	*fis/vsr*	*PccS1_04001*	—	−3.54	−4.61	−2.67	—	−2.84	−3.97	—
σ‐54 transcriptional regulator	*fis/yfhA*	*PccS1_04338*	—	—	—	—	—	—	—	—
Histone family, nucleoid‐structuring H‐NS	*hns*	*PccS1_00634*	—	—	—	—	—	—	—	—
Nutrient starvation response	*hns*	*PccS1_02657*	−2.50	—	—	—	—	—	—	—
DNA‐binding transcriptional dual regulator	*hns*	*PccS1_02662*	−3.25	—	—	−2.67	−2.80	—	—	−2.27
Histone family, nucleoid‐structuring H‐NS	*hns*	*PccS1_03336*	−**4.41**	—	−3.47	−4.27	−**3.08**	—	−2.11	−2.98
DNA‐binding transcriptional dual regulator	*crp*	*PccS1_00795*	−2.82	—	—	−2.39	−2.49	—	—	−2.11
DNA‐binding transcriptional regulator	*hexR*	*PccS1_03188*	−2.31	—	−2.53	−2.16	−2.19	—	−2.39	−2.08
DNA‐binding transcriptional regulator	*hexA*	*PccS1_04093*	—	−2.39	−2.64	−3.39	—	—	−2.10	−2.92

**FIGURE 5:**
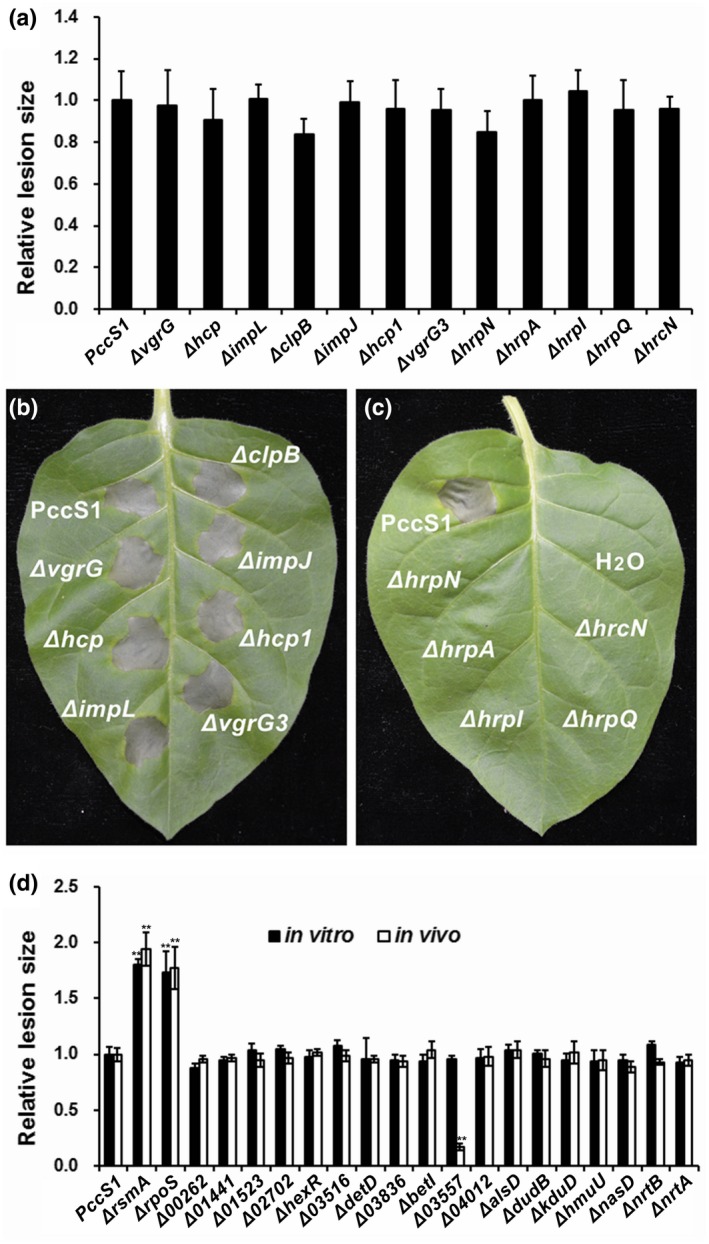
Virulence in the host plants and hypersensitive response in nonhost plants inoculated with *Pectobacterium* PccS1 and the mutants. (a) Bars represent the relative maceration areas caused by PccS1 and the strains with a mutation in a gene from the T6SS and T3SS in the detached petioles of *Brassica rapa* subsp. *pekinensis* plants 16 hr after inoculation. (b) and (c) Hypersensitive response in *Nicotiana tabacum* 'Samsun' leaves infiltrated with PccS1 and the strains with a mutation in a gene from the T6SS and T3SS, sterilized water as a negative control. Photographs were taken 24 hr after infiltration. (d) Bars represent the relative maceration areas caused by the wild‐type PccS1 and the strains (with a mutation in a PccS1 gene significantly differentially expressed when grown in planta versus those in media) in the petioles of *Zantedeschia odorata* plants in vivo and in vitro*.* Maceration areas caused by PccS1 were set to 1 to calculate the relative maceration ratio caused by the mutants. The assays were performed three times with at least three internal replicates in each. The results are shown as averages ± *SD*. **p < .01, versus that caused by the wild type, Duncan's multiple range test

The results revealed that the expression patterns of the seven genes related to construction of RNA are different (Box 1 in Table 4): *rsmA* (*PccS1_00073* and *PccS1_00521*), *rsmB* (*PccS1_00733* and *PccS1_02891*), *rsmC* (*PccS1_01829* and *PccS1_01902*), and *rpoS* (*PccS1_00230*). It is well known that RsmA is an RNA‐binding protein responsible for response to bacterial metabolic status (Charkowski *et al.*, 2012). It negatively regulates PCWDE production and represses tissue maceration (Vakulskas *et al.*, 2015). The expression pattern of *rsmA* (*PccS1_00073*) suggests that RsmA might be a switch for cellular processes including infection. The result that the *rsmA* deletion mutant showed an increase in both virulence (Figure 5d) and PCWDE activities (Figure S3) supports previous work (Vakulskas *et al.*, 2015). It has been demonstrated that SREs possess the *rpoS* gene to produce the alternate σ factor, and that *rpoS* ^−^ strains are more sensitive to hydrogen peroxide, carbon starvation, and acidic pH, and produce more PCWDEs (Mukherjee *et al.*, 1998). Similarly, our results revealed that *rpoS* in PccS1 in planta was down‐regulated (Table 4) and the virulence of Δ*rpoS* on *Z. odorata *in vitro and in vivo was increased (Figure 5d). This confirms that *rpoS* negatively regulates PCWDE production in PccS1, which is validated by the PCWDE activity assays (Figure S3).

The expression levels of the genes for two key regulators, KdgR (*PccS1_03240*) and ExpR (*PccS1_01370*), were nearly the same as for the controls, except there was less down‐regulation of *expR* at the final stage of infection compared to the control in LB only. Similarly, the expression of *carI* showed no difference compared with the references, although it was repressed at a lower level in the PccS1 samples recovered at 12 and 16 HAI compared with the reference from MM (Table 4). In our recent work, it was found that the expression of *kdgR* in the PccS1 Δ*hfq* mutant, which had completely lost maceration ability, was the same as the wild type at the transcriptional and translational level (Wang *et al*., 2018a). Here, we deduced that the comparable expression pattern of *kdgR* and *carI* might be because their expression is similar regardless of cellular processes due to their global regulatory function.

Of the four genes for sensing nitrate/nitrite or nitrogen metabolism (*narX*‐*narL* and *ntrC*‐*ntrB*), three were differentially expressed based on nutrient levels, with gene expression levels and nutrient levels inversely correlated, while *narX* was expressed at a similar level under the three different nutrition levels (Table 4). In previous work, the genes for nitrogen metabolism (*ntrC*‐*ntrB*) were undetected, although *narX*‐*narL* expressed at a log_2_‐fold change of 1–2 (Bellieny‐Rabelo *et al.*, 2019). Together, these results emphasize the notion of diversity in nitrogen metabolism in SREs, which might relate to host adaptation.

In this work, we also found that some regulatory genes were slightly differentially expressed in PccS1 infection, including genes in response to acidic pH (*phoQ* and *phoP*), transcriptional regulation (*marR*) and anaerobic regulation (*fnf, norR,* and *arcA*). Two genes of undetected expression change (*oxyR* and *ohrR*) are linked to hydroperoxide (Table 4). These results are similar to those in *D. dadantii* and *P. carotovorum* subsp. *brasiliense* 1692 (Bellieny‐Rabelo *et al.*, 2019). Together with the expression changes in PCWDEs, these data explore an expression programme between the key and accessory virulence determinants in SREs.

### 2.8 Expression pattern of genes encoding nucleotide‐related proteins during *Pectobacterium* PccS1 infection

Some nucleotide‐related proteins have been revealed to have regulatory function in virulence (Charkowski *et al.*, 2012; Reverchon and Nasser, 2013; Kusmierek and Dersch, 2018). The expression changes of 12 genes, including *fis*, *hns*, *crp*, *hexR*, and *hexA*, in PccS1 infection versus in the controls are listed in Box 4 in Table 4. Previous studies demonstrated that two of the regulatory proteins, H‐NS and Fis, orchestrate the topological changes of DNA to adjust the expression of many virulence factors and contribute to the temporal regulation of the virulence genes during infection in *D. dadantii* and *E. coli* (Ma *et al.*, 2013; Reverchon and Nasser, 2013; Jiang *et al.*, 2015). Our results for Fis expression pattern strongly support regulation of the virulence program in *D. dadantii*. In which changes of DNA topology are controlled by Fis and H‐NS in different pH, when *D. dadantii* establishes infection on host, a high level of Fis cellular concentration is formed to activate some factors involved in plant surface colonization, such as cellulose fibrils, biosurfactant and type IV pilus secretin/pili. DNA is supercoiled under high Fis concentration, while PCWDE production is inhibited. When Fis concentration decreases, the factors involved in colonization are repressed at the advanced stages of infection, and PCWDEs accumulate to be activated (Reverchon and Nasser, 2013). Our data reflect this programme with gene expression changes (Tables S1.2 and S1.3) and show that the expressions of *crp*, *hexR*, and *hexA* were all negatively regulated in planta versus the references (Box 4 in Table 4). CRP, the cyclic AMP receptor protein, has been identified as the regulator of the pectinolysis gene in *D. dadantii* (Nasser *et al.*, 1997), and as a transcriptional master regulator of numerous noncoding RNAs in the regulatory architecture linking nutritional status to virulence in *Yersinia pseduotuberculosis* (Nuss *et al*., 2015). Transcriptional factor HexR has been characterized as a global regulator of the central carbohydrate metabolism genes in various groups of proteobacteria (Leyn *et al.*, 2011). In our recent work, it was demonstrated that the expression of *hexA* was significantly increased at the transcriptional and translational level in the absence of *hfq*, which revealed the negative role of *hexA* in regulating the virulence of PccS1 (Wang *et al*., 2018a), similar to previous reports (Mukherjee *et al.*, 2000; Tobias *et al.*, 2017) (Table 4).

The 33 genes in the genome having amino acid motifs associated with cyclic nucleotide metabolism and binding are listed in Table S2. They encode proteins in categories, including catalytic and regulatory domains of diguanylate phosphodiesterases, diguanylate cyclases, and cyclic di‐GMP regulator. One third of these genes were down‐regulated in the course of infection, and only three genes were temporarily up‐regulated (two at the initial stage and one at successful infection stage) compared with the controls. Our data support previous research that showed that constitutively elevated c‐di‐GMP levels are detrimental for acute infections in many animal bacterial pathogens, such as *Vibrio cholerae* and *Brucella melitensis* (Romling *et al.*, 2013), although the molecular regulatory mechanisms underlying c‐di‐GMP are awaiting further study in *Pectobacterium*.

### 2.9 Expression pattern of the genes encoding the components for the secretion systems related to pathogenicity

It is well known that several secretory systems in bacterial pathogens secrete enzymes or toxins into host cells or the surrounding environment (Izore *et al.*, 2011; Douzi *et al.*, 2012; Koo *et al.*, 2012; Bondage *et al.*, 2016). Genes annotated in the genome of PccS1 were checked for components of the secretory systems. Both in the cluster and in the solitary loci outside the cluster, there were 25 genes encoding for the T2SS, 36 genes for the T3SS, and 29 genes for the T6SS, as well as the gene for the T3SS effector (*dspE*), the related genes (*dspF*), and a gene for the chaperone of HrpW. The gene expression pattern showed that the clusters of both T3SS and T6SS were all significantly up‐regulated in planta compared with the references (boxes in Tables S3 and S4), as were most of the genes outside the T6SS cluster (Table S4). These results are similar to those for *D. dadantii* and *P. carotovorum* subsp. *brasiliense* 1692 (Pédron *et al*., 2018; Bellieny‐Rabelo *et al.*, 2019). Meanwhile, four (*tadA, B, C* and *rcpA*) out of the 11 genes outside the T2SS cluster were expressed at higher levels when nutrient levels decreased, and the 14 genes in the T2SS cluster expressed at a similar level to the references, except six of them were up‐regulated at a log_2_‐fold ratio of 2–3 versus the LB reference (Table S5), again similar to that in *D. dadantii* (Pédron *et al*., 2018). The T2SS is known as the out system, and is responsible for secreting PCWDEs in most SRE pathogens (Charkowski *et al.*, 2012). Based on the characterization of T2SS assembly and the model of exoprotein delivery (Douzi *et al.*, 2012), the expression pattern of the T2SS genes in PccS1 recovered after inoculation indicated that the genes for the Tat export pathway (*tadB, C*) and motility related to the pilus assembly crossing the inner membrane (*tadA* and *rcpA*) were more susceptible to nutrient conditions than those in the cluster encoding Gsp proteins for assembling the secretion tunnel. We deduced that the T2SS secretion tunnel might be assembled for secretion at any cellular processes regardless of growth conditions in *Pectobacterium* PccS1. The genes with functions for pathogenicity, including those for the Tat export pathway and pili for motility in T2SS, are activated only in the presence of host plants.

### 2.10 Expression pattern of the genes for the enzymes in carbon metabolic pathways

**FIGURE 4:**
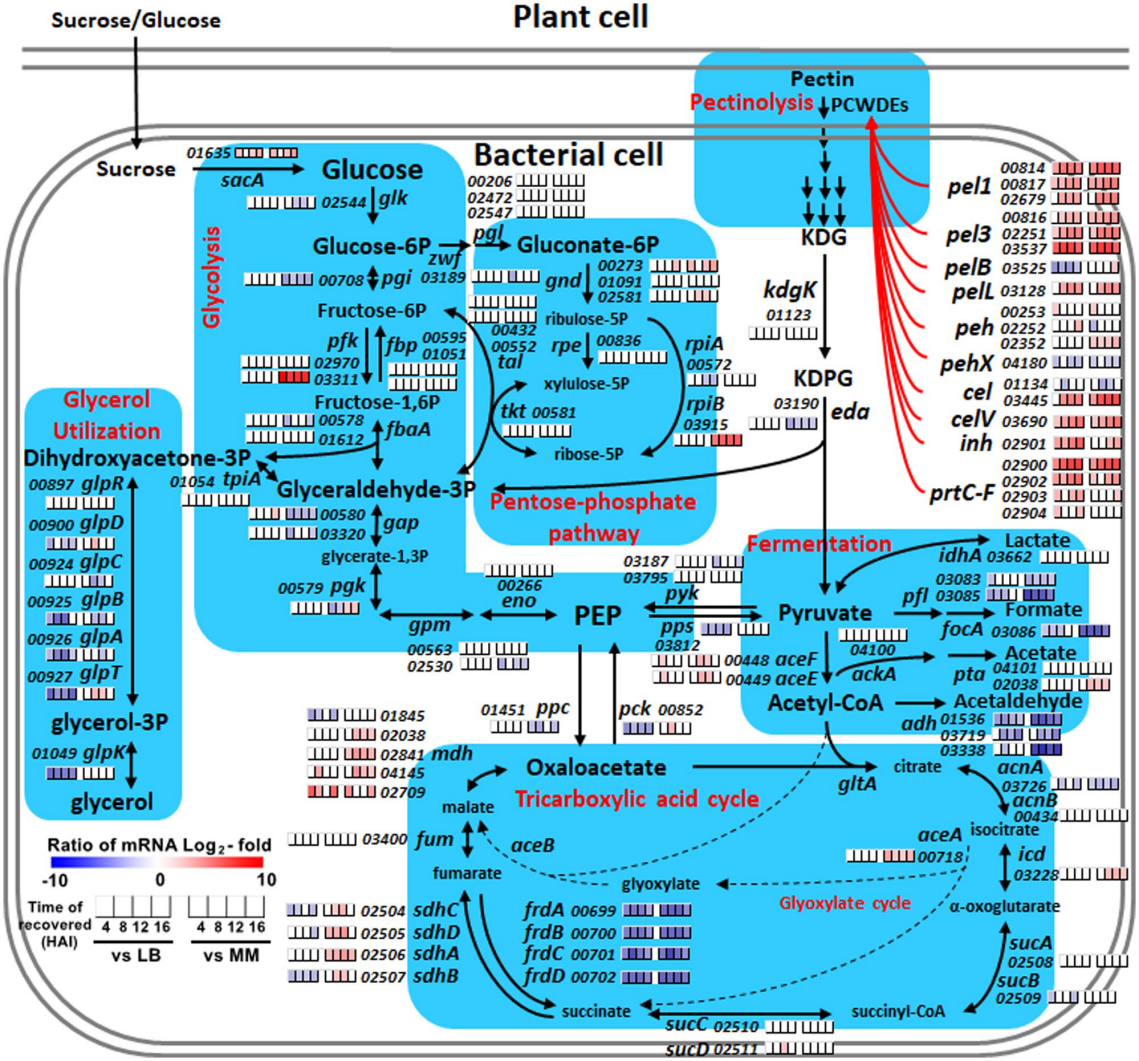
Spatiotemporal expression pattern of the genes encoding the enzymes in carbon metabolic pathways in *Pectobacterium* PccS1 recovered after inoculation at four time points versus that of the cells grown in Luria Bertani medium (LB) and minimal medium (MM)

It is well known that pectinolysis is carried out by several pectinolytic enzymes in SRE (Hugouvieux‐Cotte‐Pattat *et al.*, 1996; Joshi *et al*., 2016b). Glucose is produced in plants and converted into sucrose transported in a sieve tube. Sucrose/glucose could be utilized as nutrients when PccS1 was inoculated into the petioles of *Z. odorata* plants through a wound (Truesdell *et al.*, 1991). We summarize the expression pattern of the genes involved in the pathways of pectinolysis and carbohydrate metabolism in Figure 4, and the genes for the PCWDEs differentially expressed at a log_2_‐fold ratio over 2 versus the references are discussed in Table 3. It is notable that expression of *sacA*, *pfk*, and *rpiB* were significantly activated in the presence of sucrose or glucose, similar to the activated PCWDEs in infection, although they belong to different types of gene expression. Based on the spatiotemporal expression pattern of the genes for the enzymes in the tricarboxylic acid cycle, fermentation, and the glyoxylate cycle, presented in Figure 4, it is suggested that the metabolic pathways from sucrose/glucose and pectin to acetyl‐CoA via pyruvate are the main carbon metabolism pathways in PccS1 during infection, and these pathways might be subsequently pushed by the interconversion between succinate and fumarate (Figure 4). Meanwhile, the expression of the genes for the enzymes in other pathways (pyruvate fermentation to produce lactate, acetate, and formate, and conversion to phosphoenolpyruvate) rarely changed in any the infection samples compared to the controls (Figure 4). Previous research has indicated that the limitation of acid‐production from pyruvate is helpful to activate PCWDE production (Reverchon and Nasser, 2013). Thus, we can deduce that the substrates taken from the host by PccS1 are mainly metabolized to produce more energy and suitable to the establishment of infection through PCWDE activation.

### 2.11 Virulence of the strains with a mutation in one of the sDEGs

To further understand the functions of the sDEG in the course of infection on *Pectobacterium* PccS1 virulence, 33 sDEGs were selected for single gene deletion, and the virulence of the mutants was determined in *Brassica rapa* subsp. *pekinensis* and *Z. odorata* plants. The results indicated that the effects of the sDEGs on PccS1 virulence are different (Figure 5a,d).

Five strains with a mutation in a gene for T6SS effectors, regulation, and structure (*vgrG*s, *hcp*s, *impL*, *clpB*, and *impJ*) presented a similar level of maceration on the hosts as the wild type (Figure 5a). These results are different to those for some plant and animal pathogens that use T6SS as a weapon to interact with the hosts, such as *Acidovorax citrulli* AAC00‐1 (Tian *et al.*, 2015), *Erwinia amylovora* NCPPB1665 (Tian *et al.*, 2017), *Pantoea ananatis* LMG 2665^T^ (Shyntum *et al.*, 2015), *Pectobacterium atrosepticum* SCRI1043 (Bell *et al.*, 2004; Liu *et al.*, 2008), *Pseudomonas aeruginosa* PAO1 (Hachani *et al.*, 2016), *Vibrio cholera* V52 (Miyata *et al.*, 2011), and *Burkholderia pseudomallei* E8 (Hopf *et al.*, 2014). Our results agree with those from *E. amylovora* CFBP1430 (Kamber *et al.*, 2017) and *P. atrosepticum* SCRI1043 (Mattinen *et al.*, 2007), which showed no differences in lesion development and nonhost hypersensitive response elicited between the mutants and the wild type (Figure 5a,b). It is well known that VgrG and Hcp act as both effector and structural protein in the T6SS in pathogenic bacteria (Basler *et al.*, 2012; Ho *et al.*, 2014). Significantly activated expression of these genes for the components of T6SS in *Pectobacterium* PccS1 in planta (Table S4) revealed that these genes in the T6SS in PccS1 might participate in pathogenicity indirectly. How the T6SS in PccS1 affects virulence requires further work to scan each T6SS component.

The strains with a mutation in one of the T3SS structural and regulatory genes (*hrpN*, *hrpA*, *hrpI*, *hrpQ*, and *hrcN*) showed no differences in lesion development compared with the wild‐type PccS1 (Figure 5a), although they could not elicit a nonhost plant hypersensitive response (Figure 5c). This is different to the previous reports that *P. carotovorum* WPP14 used T3SS to induce plant cell death to promote leaf maceration in *Nicotiana benthamiana* and *Erwinia chrysanthemi* in African violet varieties (Yang *et al.*, 2002; Kim *et al.*, 2011). The results in Figure 5 and Table S3 show that *Pectobacterium* PccS1 uses T3SS to induce plant cell death rather than to macerate plant tissue directly. This strongly supports the statement that the strains naturally lacking a T3SS use other genes to compensate during attack of potato stems or tubers (Charkowski *et al.*, 2012).

The increased virulence of the strains with a mutation in *rsmA* (*PccS1_00073*) or *rpoS* (Figure 5d) confirms the function of the negative regulators, similar to previous work (Vakulskas *et al.*, 2015), and also agrees with the significantly depressed expression of these genes observed in the transcriptome profiles of PccS1 in planta versus the in vitro controls (Table 4).

**FIGURE 6:**
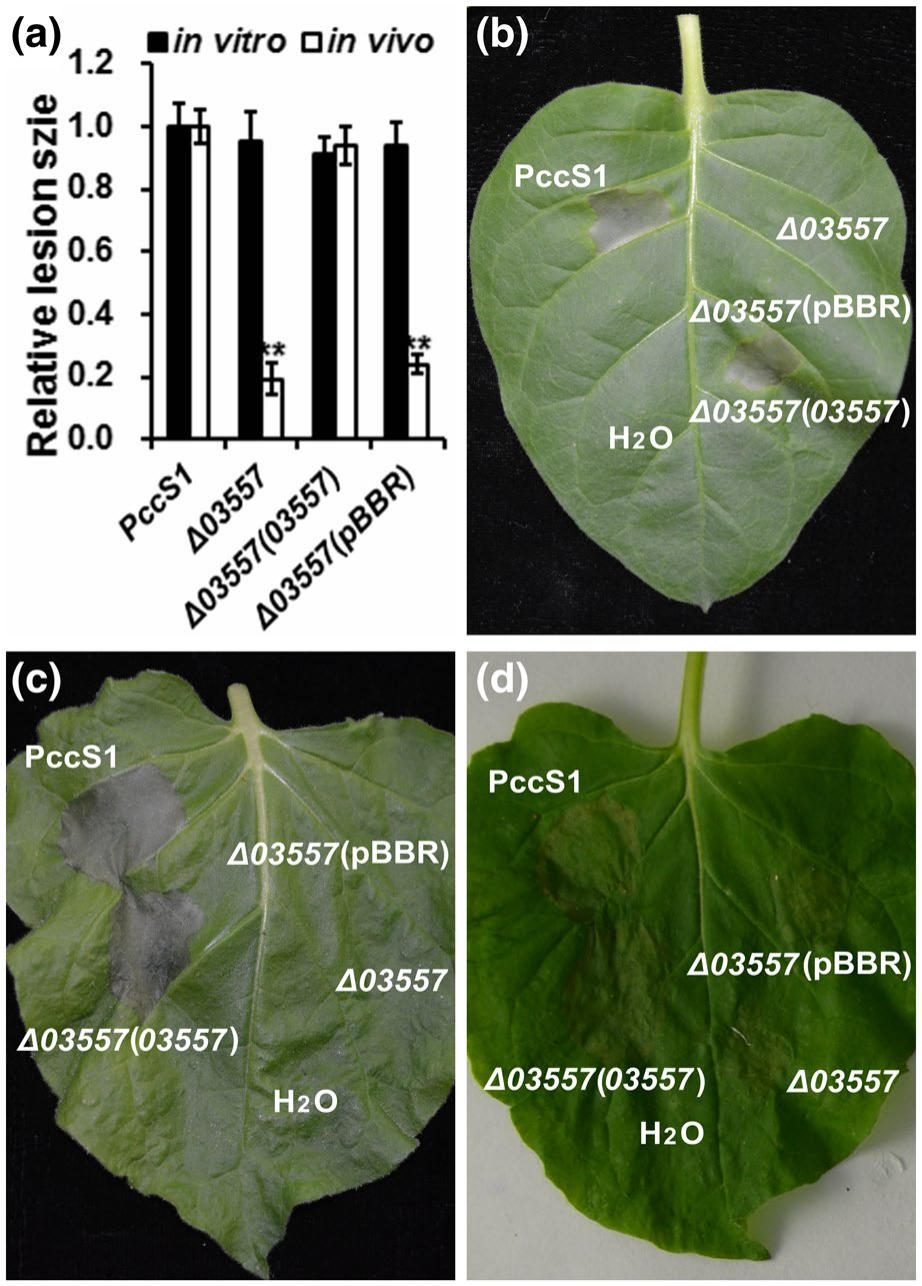
Virulence in the host plants and hypersensitive response in nonhost plants infiltrated with the bacterial strains. (a) Bars represent the relative maceration areas caused by *Pectobacterium* PccS1, gene deletion mutant Δ*03557*, the strains of Δ*03557* complemented with the plasmid carrying the fragment of *PccS1_03557*, and the empty vector in the petioles of *Z. odorata* plants in vitro and in vivo. Data are shown as averages ± *SD*. **p < .01, Duncan's multiple range test. Images show the hypersensitive response in *Nicotiana tabacum* 'Samsun' leaves in vivo (b), *N* *icotiana* *benthamiana* leaves in vivo (c) and in vitro (d) 24 hr after infiltration with the bacterial strains, and sterile water as a negative control. The assays were performed three times with at least three internal replicates in each

**FIGURE 7:**
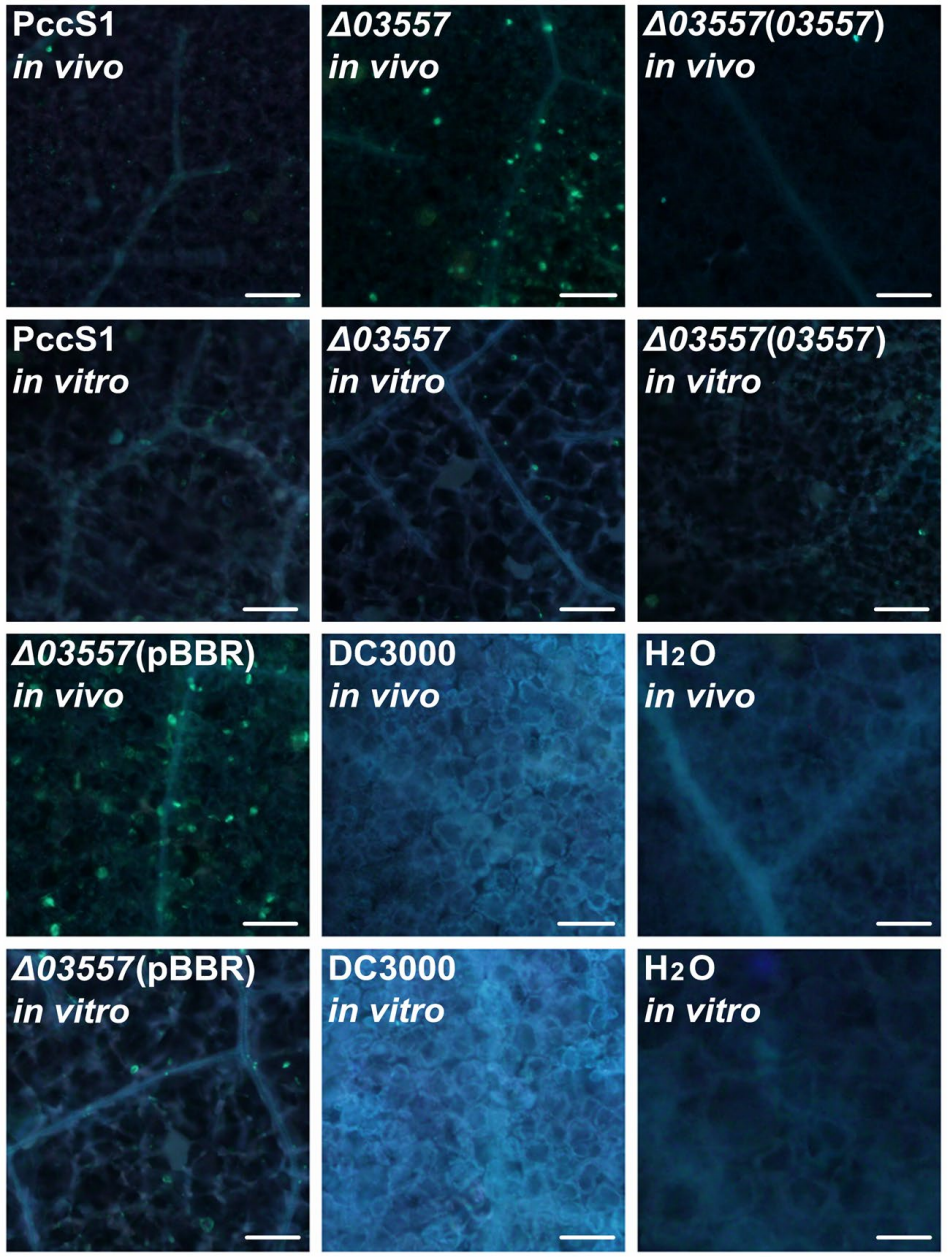
Callose deposition in *Nicotiana benthamiana* leaves in vivo and in vitro infiltrated with the bacterial strains. The images show portions of the tobacco leaves stained with aniline blue at 12 hr after bacterial infiltration. PccS1, the wild type; Δ*03557*, the deletion mutant; Δ*03557*(*03557*) and Δ*03557*(pBBR), the strains of Δ*03557* complemented with the plasmid carrying the fragment of *PccS1_03557* and the empty vector, respectively. *Pseudomonas syringae* pv. *tomato* DC3000 and sterile water were used as controls. The experiments were repeated three times with at least three internal replicates in each

Interestingly, the strain with a mutation in sDEG *PccS1_03557*, a gene encoding a two‐component transcriptional regulator belonging to the LuxR family, showed remarkable attenuated virulence in the hosts in vivo, although it macerated the plants in vitro similar to the other strains with a mutation in one of the sDEGs that caused maceration in the *Z. odorata* petioles in vitro and in vivo at the wild‐type level (Figure 5d). A role for this gene has not been shown to be differentially expressed in work on *D. dadantii* and *Pcd*1692 (Pédron *et al*., 2018; Bellieny‐Rabelo *et al.*, 2019). When complemented with the plasmid carrying the fragment of *PccS1_03557*, the mutant restored the maceration ability in the host in vivo, as well as the ability to elicit a hypersensitive response in both *N. tabacum* 'Samsun' and *N. benthamiana* leaves in vivo, but when complemented with the empty vector, the two phenotypes were unchanged (Figure 6a,b,c). Meanwhile, these mutants caused maceration in *N*. *benthamiana* leaves in vitro the same as the wild‐type 12 HAI (Figure 6d). The results revealed that *PccS1_03557* did not influence growth in LB medium (Figure S4). Callose rarely deposited in the in vitro* N. benthamiana* leaves infiltrated with Δ*PccS1*_*03557* and Δ*PccS1*_*03557* (pBBR) showed evidence that the leaves lost the ability of resistance to the invasion of the bacterial strains, but more callose was deposited in the leaves in vivo after inoculation of these two mutants (Figure 7). Our results indicated that *PccS1_03557* participates in depressing the plant’s ability to deposit callose for host resistance, and the deposition of callose for host resistance is a systematic process in the plant. This reveals the importance of using living plants, rather than mimics supplemented with plant extracts, in approaches for bacteria–plant interaction studies, and this was also demonstrated in our previous work (Wang *et al*., 2018b).

**FIGURE 8:**
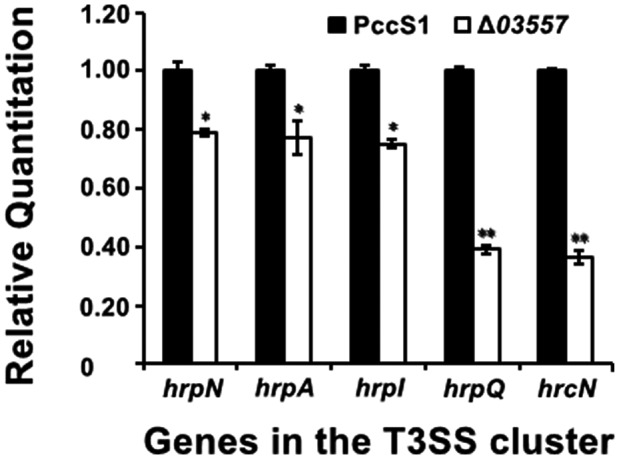
Bars represent relative mRNA levels of the genes from the T3SS in the wild‐type PccS1 and mutant Δ*03557*. The mRNA levels of these genes in PccS1 were set to 1 to calculate the relative expression ratio. The housekeeping gene *recA* was used as an endogenous control for assessing expression. The experiments were repeated three times with at least three internal replicates in each. The data are shown as averages ± *SD*. *p < .05, **p < .01 versus the wild type, Duncan's multiple range test

The results of RT‐qPCR analysis showed that the expression of T3SS structural and regulatory genes (*hrpN*, *hrpA*, *hrpI*, *hrpQ*, and *hrcN*) was down‐regulated when *PccS1*_*03557* was impaired in PccS1 (Figure 8). We have shown that the strains with a mutation in one of these genes could macerate the host at the wild‐type level and lose the ability of elicitation hypersensitive response in the leaves of the nonhost (Figure 5a,c). We deduced that the function of *PccS1*_*03557* to activate T3SS to elicit a nonhost hypersensitive response might have no relation to the function of virulence and depression of plant callose deposition for resistance. This confirms again that *Pectobacterium* PccS1 uses the T3SS to induce plant cell death rather than to macerate plant tissue. Our results revealed that maceration caused by PccS1 does not need the promotion of plant cell death elicited by the T3SS.

The present work has highlighted the advantage of gene expression experiments that recover bacterial cells after inoculation in the living plants to examine DEGs by comparing the transcriptome with that of the in vitro control not only in LB but also in MM. More DEGs were obtained from the data set of *Pectobacterium* PccS1 infection versus in MM than that of PccS1 infection versus in LB. The results of gene classification by expression pattern and the main expression pattern of genes for the enzymes in carbohydrate metabolism exhibited induction in plants and environmental adaption in *Pectobacterium* PccS1. The results also demonstrated that maceration caused by PccS1 is mainly due to the depression of plant callose deposition for resistance by the related bacterial genes and the superlytic ability of pectinolytic enzymes produced in PccS1, rather than the promotion of plant cell death elicited by the T3SS.

## 3 EXPERIMENTAL PROCEDURES

### 3.1 Bacterial strains, plasmids, and culture conditions

The bacterial strains and plasmids used in this study are listed in Table S7. Wild‐type PccS1 and the derivative strains from PccS1 were cultured in LB broth or on LB agar plate (Wang *et al*., 2018b), or MM (400 μM MgSO_4_, 7.5 mM (NH_4_)_2_SO_4_, 20 mM K_2_HPO_4_ and 15 mM KH_2_PO_4_) containing 0.5% (wt/vol) glucose (Kersey *et al.*, 2012) at 28 °C and *E. coli* on LA at 37 °C. When required, antibiotics were supplemented at the following final concentrations: kanamycin (Km) at 40 μg/ml, rifampin (Rif) at 100 μg/ml (Sangon Biotech). Bacterial growth (OD_600_) was measured in a BioPhotometer (Eppendorf) at 600 nm.

### 3.2 Assays of bacterial virulence and population dynamics in the host plants, and hypersensitive response in the nonhost plants

Virulence assays were performed as previously described (Jiang *et al.*, 2017) by inoculating appropriate bacterial suspension (OD_600_ = 1.2) onto the petioles of the approximately 45‐day‐old calla (*Z. odorata*) or *B. rapa* subsp. *pekinensis* plants in vivo or in vitro*.* Bacterial population dynamics in the host after inoculation were assayed as previously described (Andrade *et al.*, 2008; Wang *et al*., 2018b). Hypersensitive response was assayed by infiltrating bacterial strains into 6‐week‐old leaves of greenhouse‐grown tobacco plants of the two varieties (*N. tabacum* 'Samsun' and *N*. *benthamiana*) or the detached leaves of *N. benthamiana* using a needleless syringe as in previous work (Fan *et al.*, 2011; Guo *et al.*, 2012).

### 3.3 Recovery of bacterial cells from the inoculated‐plant and the media

*Pectobacterium* PccS1 were recovered using centrifugation from the petiole segments detached from the calla plants after inoculation at different time points, as previously described (Jacobs *et al*., 2012; Meng *et al.*, 2015). Each of the four sites on each 45‐day‐old petiole of the plants was inoculated with PccS1 as the virulence assay described above. The petioles were detached at 4, 8, 12, and 16 HAI, respectively, and segmented into lengths of 5 cm with the inoculation site at centre, surface sterilized with 75% ethanol, and washed three times with distilled water. The segments were longitudinally cut along the site of inoculation, then mixed with 160 ml ice‐cold transcriptional stop solution (95% ethanol, 5% water‐saturated phenol, vol/vol). Bacterial cells were harvested by centrifugation at 15,000 × g for 10 min at 4 °C. The pellets were suspended with 30 ml distilled RNase‐free water and centrifuged again at 39,000 × g at 4 °C for 20 min. After the green substances had been removed using a sterilized needle, the white pellet residues were resuspended with 2 ml RNase‐free water and centrifuged at 13,800 × g at 4 °C for 3 min. The pellets were then frozen in liquid nitrogen and stored at −80 °C for RNA extraction.

Previous research has indicated that the growth medium of the bacteria is an important consideration during the experimental design for any experimental protocol (Blair *et al.*, 2013), so we used cultures in LB and MM to set reference transcriptomic profiles for identifying differentially expressed genes in the cells grown in planta. Three millilitres of PccS1 cultures in LB or MM broth (OD_600_ = 1.0) were mixed with 3 ml ice‐cold transcriptional stop solution, and then centrifuged at 13,800 × g at 4 °C for 20 min. The pellets were frozen in liquid nitrogen and stored at −80 °C for RNA extraction.

### 3.4 RNA extraction and cDNA synthesis

RNA was extracted using an E.Z.N.A. Bacterial RNA Kit (Omega) according to the manufacturer's instructions. RNA samples were treated with RNase‐free DNase I (Thermo Fisher Scientific Inc.) to remove any DNA contamination. Purity and concentration of RNA were determined by a microspectrophotometer NanoDrop ND‐1000 (Thermo Fisher Scientific Inc.). The quality of the RNA was analysed using a 2,100 Bioanalyzer (Agilent Technologies). RNA that passed quality control was depleted using a Ribo‐Zero rRNA Removal Kit (Bacteria) (Illumina) and then broken into short fragments (approximately 200 bp). These short fragments were used as templates to synthesize cDNA libraries using the NEBNext Ultra RNA Library Prep Kit (Illumina). The cDNA libraries were purified using the Beckman AMPure XP beads (Illumina). The quality of the cDNA libraries was measured using a 2,100 Agilent High Sensitivity DNA Kit (Agilent Technologies). Quantification of the cDNA libraries was performed with an ABI 7,500 real‐time PCR system (Applied Biosystems) using a KAPA SYBR Green fast universal 2 × qPCR master mix kit (Vazyme).

### 3.5 RNA sequencing and data analysis

Pair‐end (PE) index libraries were constructed with a TruSeq PE Cluster Kit v. 3 according to the manufacturer's protocol (NEB Next Ultra Directional RNA Library Prep Kit for Illumina). The libraries with different indexes were multiplexed and loaded onto an Illumina Hiseq 2,500 according to the manufacturer's instructions (Illumina). Sequencing was carried out using a 2 × 125 paired‐end configuration. In order to obtain clean reads, raw reads were first filtered using statistical software Trimmomatic v. 0.30. Adapter sequences and low‐quality sequences were removed. Low‐quality sequences included reads of base number less than 75, reads with *N* percentage (the percentage of the nucleotides that could not be sequenced in the read) over 5%, and those with Q‐value of both the 5′ and 3′ ends lower than 20 (Q‐value of 20 means the percentages of the incorrect sequenced bases in the reads were lower than 1%). The sequences were then re‐evaluated using software FastQC v. 0.10.1 and the clean reads were assembled.

### 3.6 Identification of DEGs

The reads were mapped back to the transcriptome of PccS1 using the alignment software bowtie2 v. 2.1.0. The number of mapped clean reads for each unigene was then counted and normalized into a fragment per kilobases per million reads (FPKM) value (Mortazavi *et al.*, 2008), which was calculated by RSEM v. 1.2.4 software. Data that passed quality controls were analysed using Bioconductor edgeR software. DEGs were identified using statistical analysis among the libraries as described previously (Shen *et al.*, 2012). The *p* value threshold in multiple tests was determined by the false discovery rate (FDR) (Benjamini *et al.*, 2001; Liu *et al.*, 2015). In the present work, differentially expressed unigenes between the samples of cells recovered from living plants and cultured in LB or MM were screened with a threshold of FDR ≤ 0.05 and an absolute value of log_2_‐fold ≥ 1 as in previous studies (Liu *et al.*, 2015; Wang *et al.*, 2015; Hu *et al.*, 2016). GO and KEGG pathway enrichments were compared between up‐regulated and down‐regulated unigenes. All the unigenes were identified by BLAST comparison with the complete genome of *Pectobacterium* PccS1.

### 3.7 RT‐qPCR

To confirm the DEGs identified by RNA‐Seq in the course of PccS1 infection, RT‐qPCR assays were performed with bacterial cells collected at the time points in the processes of infection as previously described (Kersey *et al.*, 2012; Wang *et al*., 2018b). The gene‐specific primers are listed in Table S8.

### 3.8 Gene knockout and complementation

Gene knockout mutants were constructed and complemented as previously described (Wang *et al*., 2018b). Plasmids pEX18Gm carried the 300–600 bp fragment cloned from upstream or downstream of the target genes using the relevant primers (Table S6). To construct complementation strains, the target gene and its promoter region were amplified and cloned into pBBR1‐MCS5 (Table S7). All constructs were verified by PCR and sequencing.

### 3.9 Callose deposition assay

The assay of callose deposition in the leaves of *N. benthamiana* and *N. tabacum* 'Samsun' was performed as previously described (Kim *et al.*, 2011; Wang *et al*., 2018a). *P. syringae* pv. *tomato* DC3000 and sterilized water were used as controls.

### 3.10 Statistical analysis

Each assay described above was repeated at least three times with three to five replicates in each.

Data were statistically analysed using SPSS v. 14.0 (SPSS Inc.). The hypothesis test of percentages (Duncan's multiple range test, α = 0.05 or 0.01) was used to determine significant differences in the assays described above.

## CONFLICT OF INTEREST

The authors have declared no conflicts of interest.

## Supporting information


**FIGURE S1** Images of *Zantedeschia odorata* plants inoculated with *Pectobacterium carotovorum* subsp. *carotovorum* PccS1

**FIGURE S2** Bacterial population of *Pectobacterium *PccS1 recovered at different time points after inoculation in *Zantedeschia odorata* plants

**FIGURE S3** The activities of plant cell wall degrading enzymes determined for the wild type and mutants of *Pectobacterium* PccS1

**FIGURE S4** Growth curves of *Pectobacterium* *carotovorum* subsp. *carotovorum* wild type (PccS1) and the derived strains in Luria Bertani medium

**TABLE S1** The number of the differentially expressed genes (log_2_‐fold ≥ 1.0) in *Pectobacterium* PccS1 recovered from the petioles of calla lily (*Zantedeschia odorata*) plants at four different time points after inoculation compared with in vitro controls grown in both Luria Bertani and minimal media
**TABLE 1.2** Log_2_‐fold ratio (≥ 2.0) of the significantly differentially expressed genes in PccS1 recovered at four different time points after inoculation in the petioles of calla lily plants in vivo compared with the cultures in Luria Bertani medium
**TABLE 1.3** Log_2_‐fold ratio (≥ 2.0) of the significantly differentially expressed genes in PccS1 recovered at four different time points after inoculation in the petioles of calla lily plants in vivo compared with that of the cultures in minimal medium

**TABLE S2** Expression pattern of the genes encoding for cyclic nucleotide‐related proteins in *Pectobacterium* PccS1 recovered at different times after inoculation compared with that of the cells in Luria Bertani and minimal media

**TABLE S3** Log_2_‐fold ratios of the genes for the components of T3SS in *Pectobacterium* PccS1 recovered from *Zantedeschia odorata* at different times after inoculation compared with those for the cells in Luria Bertani and minimal media

**TABLE S4** Log_2_‐fold ratios of the genes in T6SS cluster and the homologues dispersed in the genome of *Pectobacterium* PccS1 recovered from *Zantedeschia odorata* at different times after inoculation compared with those of the cells in Luria Bertani and minimal media

**TABLE S5** Log_2_‐fold ratios of the genes for the components of T2SS in *Pectobacterium* PccS1 recovered from *Zantedeschia odorata* at different times after inoculation compared with those of the cells in the media

**TABLE S6** The sequences of oligonucleotides for molecular modification used in this study

**TABLE S7** Bacterial strains and plasmids used in this study

**TABLE S8** The sequences of RT‐qPCR primers used in this study

## Data Availability

The data that support the findings of this study are available from the corresponding author upon reasonable request.
