# Supplementary figures and images for: Transcriptome of Pectobacterium carotovorum subsp. carotovorum PccS1 infected in calla plants in vivo highlights a spatiotemporal expression pattern of genes related to virulence, adaptation, and host response
Source: Mol Plant Pathol. 2020 Apr 8;21(6):871–91. doi: 10.1111/mpp.12936 (PMC7214478; doi:10.1111/mpp.12936)

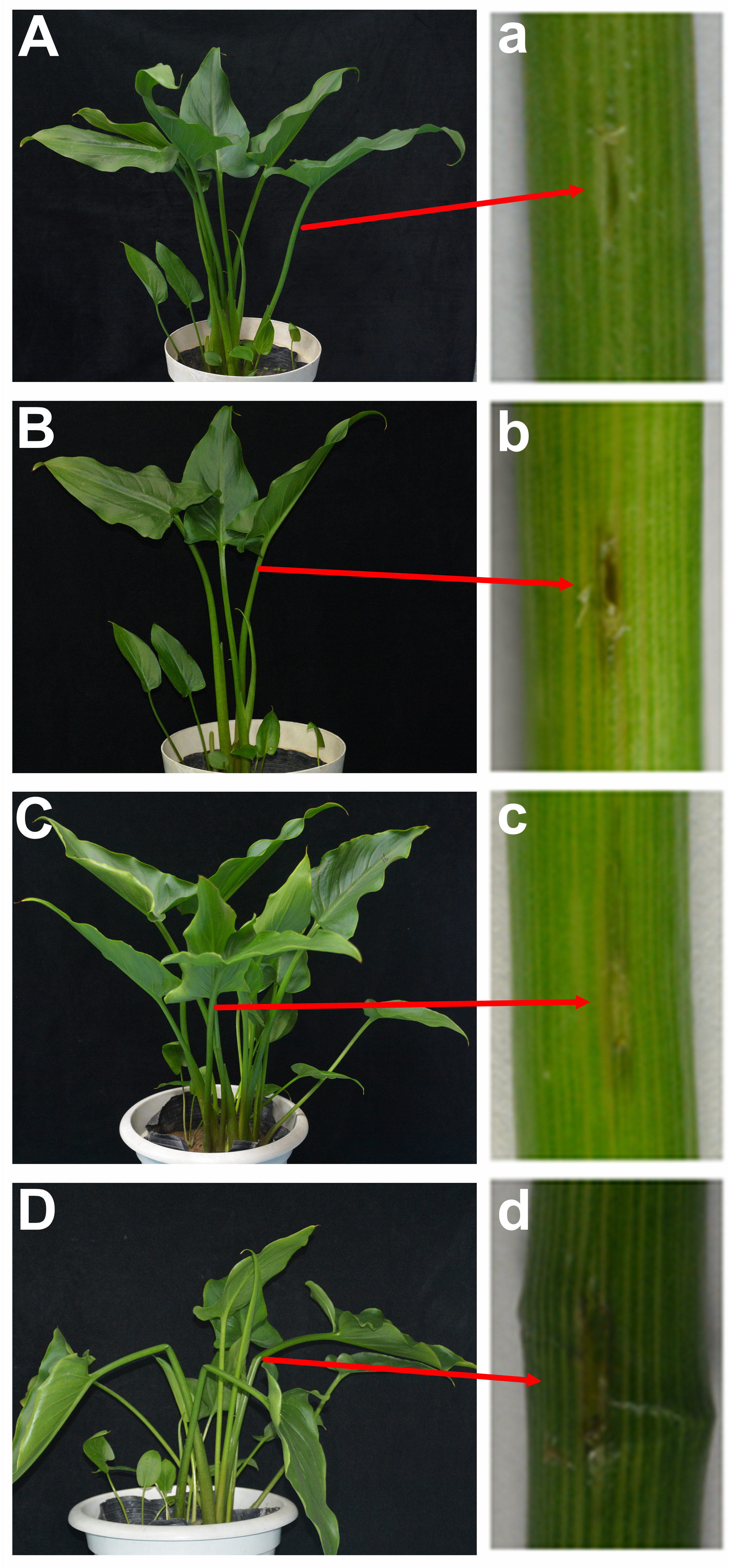

Supplement: Supplementary file 1 — FIGURE S1 Images of Zantedeschia odorata plants inoculated with Pectobacterium carotovorum subsp. carotovorum PccS1 [file MPP-21-871-s001.tiff]

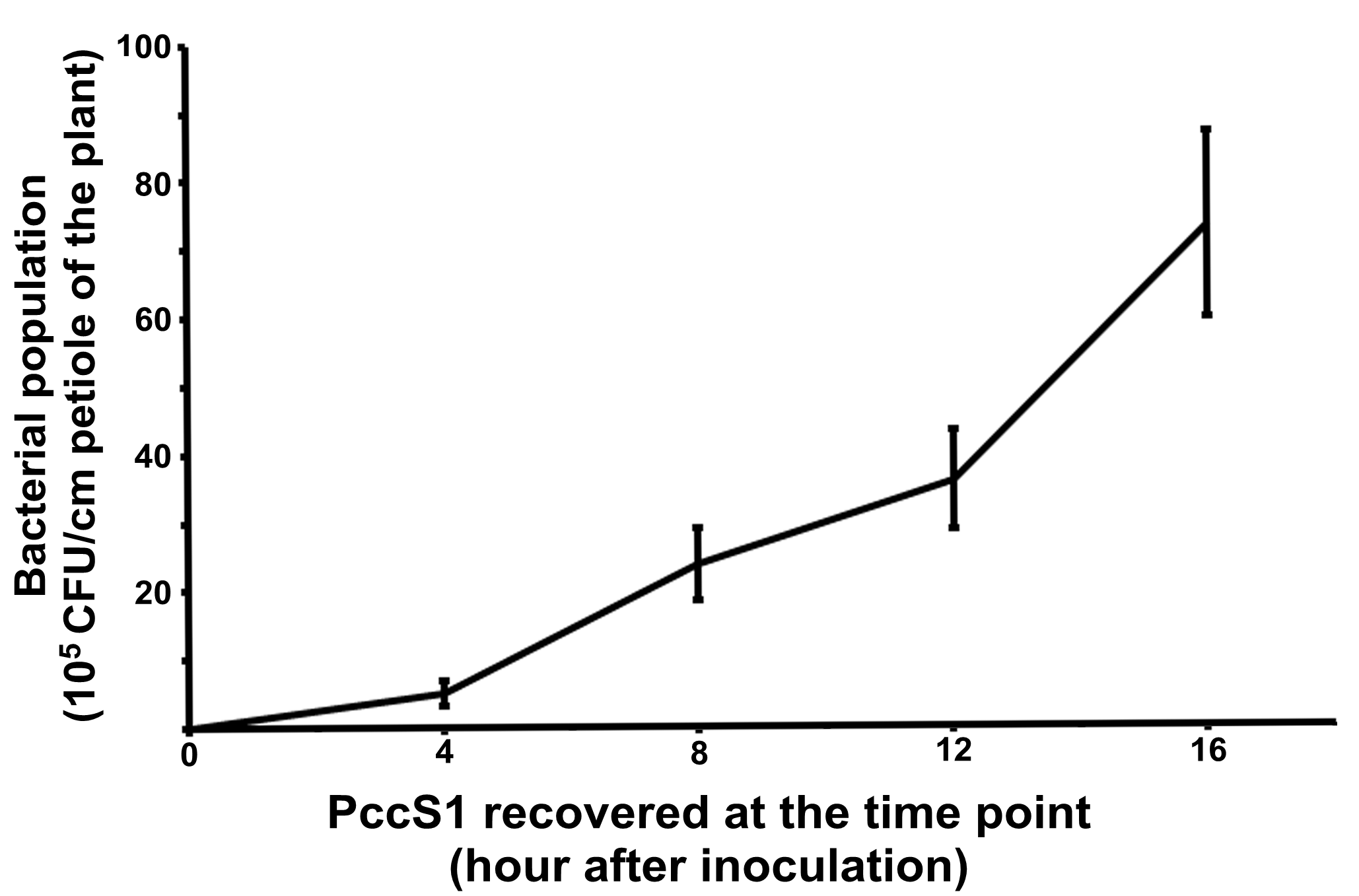

Supplement: Supplementary file 2 — FIGURE S2 Bacterial population of Pectobacterium PccS1 recovered at different time points after inoculation in Zantedeschia odorata plants [file MPP-21-871-s002.tiff]

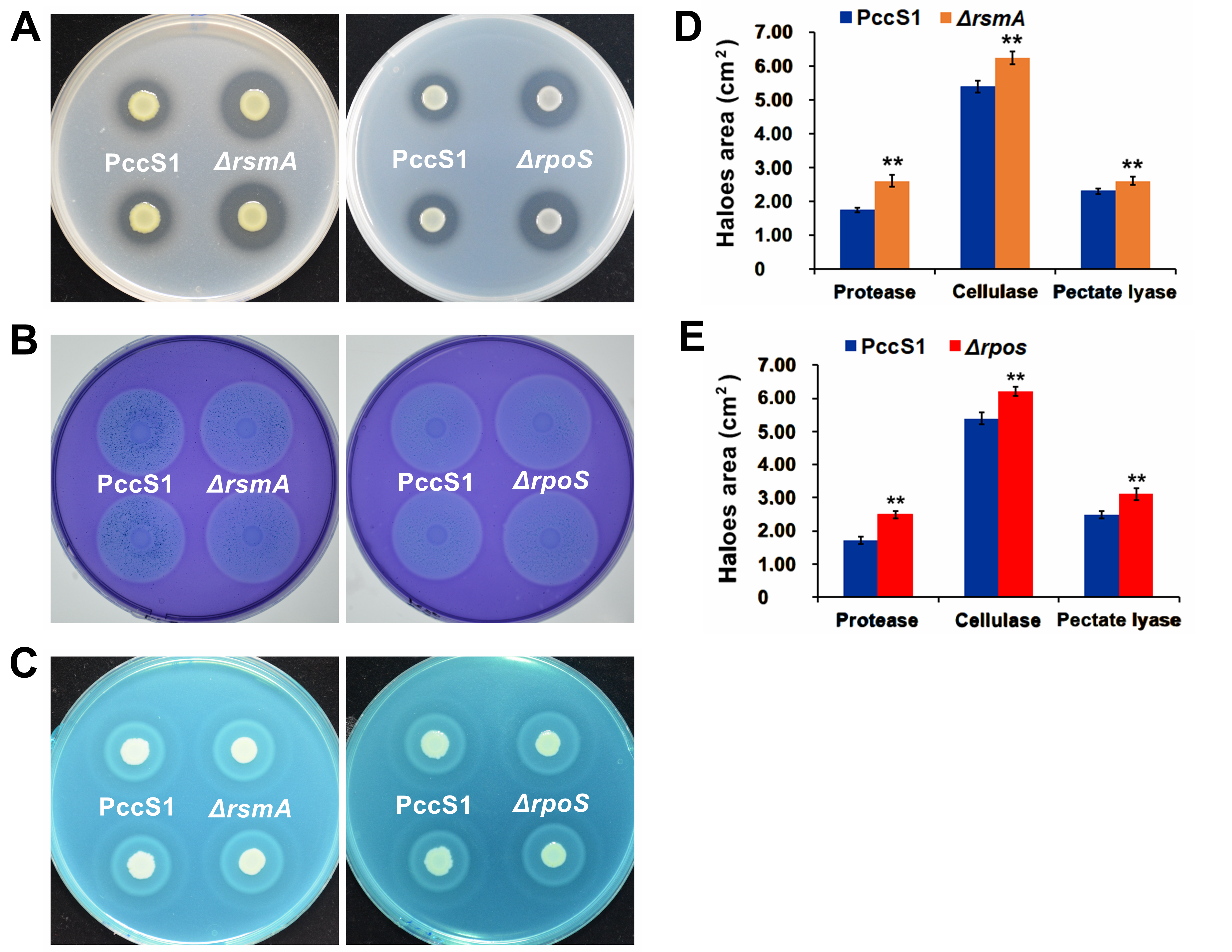

Supplement: Supplementary file 3 — FIGURE S3 The activities of plant cell wall degrading enzymes determined for the wild type and mutants of Pectobacterium PccS1 [file MPP-21-871-s003.tiff]

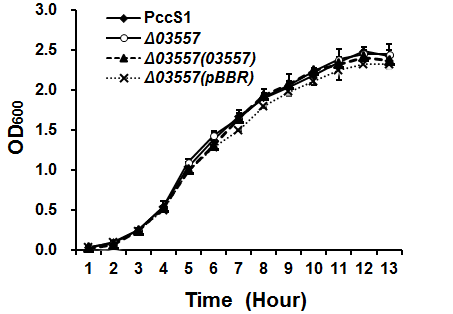

Supplement: Supplementary file 4 — FIGURE S4 Growth curves of Pectobacterium carotovorum subsp. carotovorum wild type (PccS1) and the derived strains in Luria Bertani medium [file MPP-21-871-s004.tiff]
