# Supplementary material for: Transcriptome of Pectobacterium carotovorum subsp. carotovorum PccS1 infected in calla plants in vivo highlights a spatiotemporal expression pattern of genes related to virulence, adaptation, and host response
Source: Mol Plant Pathol. 2020 Apr 8;21(6):871–91. doi: 10.1111/mpp.12936 (PMC7214478; doi:10.1111/mpp.12936)
Supplement: Supplementary file 6 — TABLE S2 Expression pattern of the genes encoding for cyclic nucleotide‐related proteins in Pectobacterium PccS1 recovered at different times after inoculation compared with that of the cells in Luria Bertani and minimal media [file MPP-21-871-s006.docx]

**Table S2** Expression pattern of the genes encoding for cyclic nucleotide-related proteins in PccS1 recovered at different time after inoculation compared with that of the cells in the media of LB and MM

| **Function**  **(annotated with multi-databases)** | **amino caid motif** | **Gene ID** | **Ratio of log_2_-fold (vs. that in LB)** | | | |  | | **Ratio of log_2_-fold (vs. that in MM)** | | | | |  |
| --- | --- | --- | --- | --- | --- | --- | --- | --- | --- | --- | --- | --- | --- | --- |
|  |  |  | **4 h** | **8 h** | **12 h** | **16 h** | |  | | **4 h** | **8 h** | **12 h** | **16 h** | |
| diguanylate phosphodiesterase | EAL | *PccS1_0249* | — | — | — | — | |  | | 2.54 | — | — | — | |
| EAL domain-containing protein | EAL | *PccS1_1310* | — | — | — | — | |  | | — | — | — | — | |
| EAL domain-containing protein | EAL | *PccS1_2239* | — | — | — | — | |  | | — | -2.59 | — | -2.15 | |
| **diguanylate phosphodiesterase** | **EAL** | ***PccS1_3169*** | **-2.27** | **-3.99** | **-4.28** | **-5.71** | |  | | **-2.26** | **-3.87** | **-4.24** | **-5.73** | |
| EAL domain-containing protein | EAL | *PccS1_3555* | — | — | — | — | |  | | — | — | — | — | |
| diguanylate cyclase/phosphodiesterase | EAL | *PccS1_3654* | — | — | — | — | |  | | — | — | — | — | |
| diguanylate phosphodiesterase | EAL | *PccS1_3820* | — | — | — | — | |  | | — | -2.01 | -2.57 | — | |
| cyclic di-GMP phosphodiesterase | EAL | *PccS1_4354* | — | — | — | — | |  | | — | — | — | — | |
| diguanylate cyclase/phosphodiesterase | EAL/GGDEF | *PccS1_1537* | — | — | — | — | |  | | — | -3.03 | -2.47 | -2.23 | |
| PAS/PAC and GAF sensor-containing diguanylate cyclase/phosphodiesterase | EAL/GGDEF | *PccS1_3232* | — | — | -2.23 | — | |  | | -2.27 | -2.26 | -2.65 | -2.40 | |
| diguanylate cyclase/phosphodiesterase with extracellular sensor | EAL/GGDEF | *PccS1_4347* | — | — | — | — | |  | | — | — | — | — | |
| diguanylate cyclase | GGDEF | *PccS1_0081* | — | — | — | — | |  | | — | -3.54 | -3.17 | -3.45 | |
| PAS/PAC and GAF sensor-containing diguanylate cyclase | GGDEF | *PccS1_0328* | — | — | — | — | |  | | — | -2.11 | — | — | |
| diguanylate cyclase (precursor) | GGDEF | *PccS1-0513* | — | — | — | — | |  | | — | — | — | — | |
| diguanylate cyclase | GGDEF | *PccS1_0551* | — | — | — | — | |  | | -2.39 | — | -2.48 | — | |
| diguanylate cyclase | GGDEF | *PccS1_1126* | — | — | — | — | |  | | — | — | — | — | |
| diguanylate cyclase | GGDEF | *PccS1_1227* | — | — | — | — | |  | | — | — | — | — | |
| diguanylate cyclase | GGDEF | *PccS1_1833* | — | — | — | — | |  | | 2.21 | — | — | — | |
| diguanylate cyclase | GGDEF | *PccS1_2244* | — | — | — | — | |  | | — | — | 2.76 | — | |
| diguanylate cyclase | GGDEF | *PccS1_2590* | — | — | — | — | |  | | — | — | — | — | |
| diguanylate cyclase | GGDEF | *PccS1_2832* | — | — | — | — | |  | | — | — | — | — | |
| diguanylate cyclase | GGDEF | *PccS1_3975* | — | — | — | — | |  | | — | — | — | — | |
| **diguanylate cyclase** | **GGDEF** | ***PccS1_4218*** | **—** | **-3.32** | **-2.17** | **-2.50** | |  | | **—** | **-3.11** | **2.03** | **-2.42** | |
| diguanylate cyclase | GGDEF | *PccS1_4269* | — | — | — | -2.40 | |  | | — | -2.05 | -2.36 | -2.98 | |
| PAS/PAC and GAF sensor-containing diguanylate cyclase | GGDEF | *PccS1_4353* | — | — | -3.16 | -3.37 | |  | | — | — | -2.74 | -3.02 | |
| polynucleotide adenylyltransferase | HD-GYP | *PccS1_00290* | — | — | — | — | |  | | — | — | — | — | |
| UTP-GlnB uridylyltransferase | HD-GYP | *PccS1_2204* | — | — | — | — | |  | | — | — | — | — | |
| metal-dependent phosphohydrolase | HD-GYP | *PccS1_3490* | — | — | — | — | |  | | — | — | — | — | |
| deoxyguanosinetriphosphate triphosphohydrolase | HD-GYP | *PccS1_4386* | — | — | — | — | |  | | — | — | — | — | |
| putative metal dependent phosphohydrolase | PilZ | *PccS1_0248* | — | — | — | — | |  | | — | — | — | — | |
| cellulose synthase catalytic subunit | PilZ | *PccS1_1132* | — | -2.57 | — | — | |  | | — | -2.90 | — | — | |
| cyclic di-GMP binding protein | PilZ | *PccS1_3113* | — | — | — | — | |  | | — | — | — | -2.05 | |
| cyclic di-GMP regulator |  | *PccS1_1133* | — | -2.47 | — | — | |  | | — | -2.64 | — | — | |
