# Supplementary material for: Transcriptome of Pectobacterium carotovorum subsp. carotovorum PccS1 infected in calla plants in vivo highlights a spatiotemporal expression pattern of genes related to virulence, adaptation, and host response
Source: Mol Plant Pathol. 2020 Apr 8;21(6):871–91. doi: 10.1111/mpp.12936 (PMC7214478; doi:10.1111/mpp.12936)
Supplement: Supplementary file 7 — TABLE S3 Log2‐fold ratios of the genes for the components of T3SS in Pectobacterium PccS1 recovered from Zantedeschia odorata at different times after inoculation compared with those for the cells in Luria Bertani and minimal media [file MPP-21-871-s007.docx]

**T****able S3** Log_2_-fold ratios of the genes for the components of T3SS in *Pectobacterium* PccS1 recovered from *Zantedeschia odorata* at different time after inoculation compared with that of the cells in the media of LB and MM

| **Function** | **Gene Name** | **Gene ID** | **Ratio of log_2_-fold (vs that in LB)** | | | |  | **Ratio of log_2_-fold (vs that in MM)** | | | |
| --- | --- | --- | --- | --- | --- | --- | --- | --- | --- | --- | --- |
|  |  |  | **4** | **8** | **12** | **16** |  | **4** | **8** | **12** | **16** |
| Hrp dependent T3SE |  | *P**ccS1_00416* | 3.13 | — | — | 2.26 |  | 2.78 | — | — | — |
| Putative T3SE Hop protein |  | *PccS1_00661* | — | — | — | — |  | — | — | — | — |
| T2SS/T3SS, GspD/PilQ family | *pilQ* | *PccS1_00841* | 2.22 | — | — | — |  | — | — | — | — |
| Hrp dependent T3SE | *ygbK* | *PccS1_01092* | — | — | — | — |  | 2.60 | — | — | — |
| T3SS flagellar brake protein | *ycgR* | *PccS1_03113* | — | — | — | — |  | — | — | — | -2.05 |
| Avirulence protein | *dspF* | *PccS1_03535* | 3.30 | 4.21 | 4.46 | 3.05 |  | 4.56 | 5.59 | 5.76 | 4.27 |
| Putative avirulence protein | *dspE* | *PccS1_03536* | 5.57 | 5.51 | 5.94 | 5.45 |  | 5.67 | 5.72 | 6.07 | 5.52 |
| Potential HrpW-specific chaperone |  | *PccS1_03538* | 4.89 | 5.85 | 5.78 | 5.39 |  | 4.30 | 5.37 | 5.22 | 4.76 |
| T3SE harpin | *hrpN* | *PccS1_03544* | 8.19 | 9.66 | 9.45 | 8.67 |  | 7.12 | 8.70 | 8.41 | 7.56 |
| Type III secretion protein | *hrpV* | *PccS1_03545* | 3.60 | 3.40 | 3.90 | 3.85 |  | — | 3.24 | 3.64 | — |
| Type III secretion lipoprotein | *hrpT* | *PccS1_03546* | — | 8.66 | 9.52 | — |  | — | 7.78 | 8.34 | — |
| Outer membrane pore | *hrcC* | *PccS1_03547* | 5.23 | 5.44 | 5.83 | 5.00 |  | 5.41 | 5.73 | 6.04 | 5.15 |
| Type III secretion protein | *hrpG* | *PccS1_03548* | 7.50 | 7.64 | 7.73 | 7.13 |  | 6.12 | 6.37 | 6.37 | 5.70 |
| HrpF family | *hrpF* | *PccS1_03549* | — | 7.44 | 7.61 | — |  | — | 4.35 | 4.47 | — |
| T3SS apparatus lipoprotein | *hrpE* | *PccS1_03550* | 4.04 | 3.22 | 4.16 | 3.63 |  | 3.74 | 3.04 | 3.90 | 3.30 |
| Type III secretion protein | *hrpD* | *PccS1_03551* | 5.23 | 4.70 | 5.69 | 4.04 |  | 6.74 | 6.34 | 7.25 | 5.88 |
| T3SS apparatus lipoprotein | *hrcJ* | *PccS1_03552* | 5.42 | 4.78 | 5.44 | 4.56 |  | 6.19 | 5.67 | 6.24 | 5.29 |
| T3SS apparatus lipoprotein | *hrpB* | *PccS1_03553* | 4.60 | 4.24 | 5.11 | 2.82 |  | 6.78 | 6.56 | 7.33 | 4.96 |
| Type III secretion protein | *hrpA* | *PccS1_03554* | 4.56 | 6.94 | 6.35 | 4.61 |  | 4.81 | 7.29 | 6.63 | 4.82 |
| Sigma-factor | *hrpL* | *pccS1_03559* | 4.75 | 3.73 | 3.90 | 3.59 |  | 3.51 | 2.59 | 2.69 | — |
| Type III secretion protein | *hrpJ* | *PccS1_03560* | 8.63 | 7.91 | 8.80 | 7.80 |  | 7.24 | 6.64 | 7.45 | 6.37 |
| Type III secretion protein | *hrpI* | *PccS1_03561* | 6.26 | 5.99 | 6.69 | 5.93 |  | 6.64 | 6.49 | 7.10 | 6.27 |
| T3SS apparatus protein | *hrpQ* | *PccS1_03562* | 6.20 | 5.88 | 6.27 | 5.73 |  | 8.24 | 8.12 | 8.37 | 7.73 |
| T3SS pathway ATPase | *hrcN* | *PccS1_03563* | 6.59 | 6.25 | 6.94 | 6.29 |  | 5.86 | 5.63 | 6.25 | 5.52 |
| Type III secretion protein | *hrpO* | *PccS1_03564* | 4.85 | 3.96 | 4.61 | 5.49 |  | 5.12 | 4.37 | 4.93 | 5.72 |
| Type III secretion protein | *hrpP* | *PccS1_03565* | 3.90 | 4.44 | 4.78 | 4.47 |  | 4.62 | 5.29 | 5.53 | 5.15 |
| T3SS apparatus protein | *hrcQ/yscQ* | *PccS1_03566* | 4.09 | 4.11 | 4.47 | 3.40 |  | 5.78 | 5.92 | 6.19 | 5.06 |
| Virulence translocation | *hrcR* | *PccS1_03567* | 6.23 | 5.93 | 6.90 | 5.07 |  | 5.61 | 5.44 | 6.32 | 4.42 |
| Type III secretion protein | *hrpO* | *PccS1_03568* | — | 8.38 | 8.52 | — |  | — | 4.53 | 4.76 | — |
| Inner membrane R protein | *hrcT* | *PccS1_03569* | 5.16 | 4.28 | 5.41 | 4.41 |  | 6.54 | 5.81 | 6.84 | 5.76 |
| T3SS substrate exporter | *hrpY/yscU* | *PccS1_03570* | 3.33 | 2.61 | 3.11 | 2.78 |  | 2.54 | — | 2.34 | — |
| T3SS pathway lipoprotein/Flagellar M-ring protein | *fliF* | *PccS1_03930* | — | — | — | — |  | — | — | — | — |
| T3SS pathway protein/Flagellar assembly protein | *hrpE/fliH* | *PccS1_03932* | — | — | — | — |  | — | 2.17 | — | 2.23 |
| T3SS pathway ATPase | *fliI* | *PccS1_03933* | — | — | — | — |  | — | — | — | — |
| T3SS pathway protein/Flagellar motor switch | *hrcQb/fliN* | *PccS1_03938* | — | — | — | — |  | — | — | — | — |
| Inner membrane P protein | *fliP* | *PccS1_03940* | — | — | — | — |  | — | — | — | — |
| Inner membrane R protein | *fliR* | *PccS1_03942* | — | — | — | — |  | — | — | — | — |
| T3SS pathway chaperone | *flgN* | *PccS1_03956* | -4.37 | — | -2.81 | -2.12 |  | -4.86 | — | -3.27 | -2.65 |
| T3SS FHIPEP | *flhA* | *PccS1_03958* | — | — | — | — |  | — | — | — | — |
| T3SS substrate exporter | *flhB* | *PccS1_03959* | — | — | — | — |  | — | — | — | — |
