# Supplementary material for: Transcriptome of Pectobacterium carotovorum subsp. carotovorum PccS1 infected in calla plants in vivo highlights a spatiotemporal expression pattern of genes related to virulence, adaptation, and host response
Source: Mol Plant Pathol. 2020 Apr 8;21(6):871–91. doi: 10.1111/mpp.12936 (PMC7214478; doi:10.1111/mpp.12936)
Supplement: Supplementary file 8 — TABLE S4 Log2‐fold ratios of the genes in T6SS cluster and the homologues dispersed in the genome of Pectobacterium PccS1 recovered from Zantedeschia odorata at different times after inoculation compared with those of the cells in Luria Bertani and minimal media [file MPP-21-871-s008.docx]

**T****able S4** Log_2_-fold ratios of the genes in T6SS cluster and the homologues dispersed in the genome of *Pectobacterium* PccS1 recovered from *Zantedeschia odorata* at different time after inoculation compared with that of the cells in the media of LB and MM

| **Function** | **Gene Name** | **Gene ID** | **Ratio of log2-fold (vs that in LB)** | | | |  | **Ratio of log2-fold (vs that in MM)** | | | |
| --- | --- | --- | --- | --- | --- | --- | --- | --- | --- | --- | --- |
|  |  |  | **4** | **8** | **12** | **16** | **4** | | **8** | **12** | **16** |
|  |  |  |  |  |  |  |  | |  |  |  |
| Vgr family protein | vgrG | *PccS1_00112* | 3.81 | 5.02 | 6.29 | 7.16 | 2.34 | | 3.65 | 4.85 | 5.65 |
| T6SS effector, Hcp1 family | *hcp* | *PccS1_00113* | — | 4.10 | 5.37 | 6.00 |  | — | 4.27 | 5.47 | 6.03 |
| ImpA domain-containing protein | *vasL* | *PccS1_00115* | 2.98 | 4.03 | 4.60 | 4.49 |  | 2.88 | 4.03 | 4.53 | 4.36 |
| T6SS IcmF (precursor) | *impL/vasK* | *PccS1_00116* | 4.20 | 4.93 | 5.76 | 5.77 |  | 3.12 | 3.96 | 4.71 | 4.65 |
| Type VI secretion-associated protein | *impA/vasJ* | *PccS1_00117* | 3.52 | 4.21 | 4.79 | 5.06 |  | 4.63 | 5.44 | 5.94 | 6.14 |
| Type VI secretion-associated protein | *vasI* | *PccS1_00118* | 3.49 | 3.89 | 5.32 | 5.19 |  | 3.93 | 4.45 | 5.80 | 5.60 |
|  | *vasH* | *PccS1_00119* | 3.64 | 4.44 | 5.47 | 5.35 |  | 3.70 | 4.61 | 5.56 | 5.37 |
| Type VI secretion ATPase,  ClpV1 family | *clpB/vasG* | *PccS1_00120* | 3.16 | 4.03 | 5.03 | 5.11 |  | 3.01 | 3.99 | 4.91 | 4.92 |
| T6SS protein DotU family | *impK/vasF* | *PccS1_00121* | 3.69 | 4.79 | 5.37 | 5.05 |  | 3.36 | 4.57 | 5.07 | 4.69 |
| T6SS protein | *impJ/vasE* | *PccS1_00122* | 4.74 | 5.26 | 6.11 | 6.01 |  | 6.49 | 7.13 | 7.89 | 7.72 |
| Type VI secretion lipoprotein | *vasD* | *PccS1_00123* | 6.00 | 6.63 | 7.28 | 7.62 |  | 5.54 | 6.31 | 6.86 | 7.12 |
| Putative forkhead-associated (FHA) domain-containing protein | *impI/vasC* | *PccS1_00124* | 4.90 | 5.84 | 6.96 | 7.32 |  | 4.50 | 5.54 | 6.59 | 6.87 |
| T6SS protein | *impH/vasB* | *PccS1_00125* | 4.93 | 5.43 | 6.80 | 6.28 |  | 3.83 | 4.44 | 5.73 | 5.14 |
| T6SS protein | *impG/vasA* | *PccS1_00126* | 3.69 | 5.12 | 6.24 | 5.79 |  | 3.87 | 5.42 | 6.46 | 5.94 |
| T6SS lysozyme-related protein | *impF* | *PccS1_00127* | 3.16 | 4.54 | 5.08 | 5.51 |  | 3.84 | 5.35 | 5.80 | 6.16 |
| T6SS protein | *impC* | *PccS1_00128* | 3.27 | 5.57 | 6.10 | 5.73 |  | 4.41 | 6.82 | 7.28 | 6.84 |
| T6SS protein | *impB* | *PccS1_00129* | 4.04 | 5.64 | 5.98 | 6.41 |  | 4.70 | 6.41 | 6.67 | 7.03 |
| T6SS effector | *hcp1* | *PccS1_00132* | — | — | 2.96 | 3.15 |  | 2.25 | 2.22 | 3.30 | 3.42 |
| T6SS secreted protein VgrG | *vgrG1* | *PccS1_00181* | 2.40 | — | 2.40 | 3.05 |  | — | — | — | 2.26 |
| T6SS effector | *hcp2* | *PccS1_00387* | -3.53 | — | — | — |  | — | — | — | — |
| T6SS secreted protein VgrG | *vgrG2* | *PccS1_00685* | 3.47 | 5.09 | 6.86 | 7.74 |  | 2.73 | 4.46 | 6.15 | 6.96 |
| T6SS effector | *hcp3* | *PccS1_00686* | — | 4.75 | 6.22 | 6.75 |  | 3.30 | 6.24 | 7.63 | 8.09 |
| T6SS effector, Hcp1 family | *hcp4* | *PccS1_01154* | — | 4.09 | 5.23 | 5.28 |  | 2.74 | 5.32 | 6.38 | 6.37 |
| T6SS secreted protein VgrG | *vgrG3* | *PccS1_01155* | 3.27 | 4.74 | 5.53 | 6.72 |  | 4.75 | 6.34 | 7.05 | 8.16 |
| T6SS effector, Hcp1 family | *hcp5* | *PccS1_01437* | — | — | — | — |  | 4.01 | 3.28 | 4.25 | 3.96 |
| T6SS effector, Hcp1 family | *hcp6* | *PccS1_01748* | — | — | 2.32 | 2.08 |  | 3.48 | 2.84 | 4.05 | 3.74 |
| T6SS effector | *hcp7* | *PccS1_02233* | — | — | — | — |  | — | 2.07 | — | — |
| T6SS secreted protein VgrG | *vgrG4* | *PccS1_03542* | 3.47 |  | 2.41 | 4.30 |  | — | — | — | 2.42 |
| T6SS effector | *hcp8* | *PccS1_04193* | — | — | — | — |  | — | — | — | — |
