# Supplementary material for: Transcriptome of Pectobacterium carotovorum subsp. carotovorum PccS1 infected in calla plants in vivo highlights a spatiotemporal expression pattern of genes related to virulence, adaptation, and host response
Source: Mol Plant Pathol. 2020 Apr 8;21(6):871–91. doi: 10.1111/mpp.12936 (PMC7214478; doi:10.1111/mpp.12936)
Supplement: Supplementary file 9 — TABLE S5 Log2‐fold ratios of the genes for the components of T2SS in Pectobacterium PccS1 recovered from Zantedeschia odorata at different times after inoculation compared with those of the cells in the media [file MPP-21-871-s009.docx]

**Table S5** Log_2_-fold ratios of the genes for the components of T2SS in *Pectobacterium* PccS1 recovered from *Zantedeschia odorata* at different time after inoculation compared with that of the cells in the media

| **Function** | **Gene Name** | **Gene ID** | **Ratio of log_2_-fold (vs that in LB)** | | | |  | **Ratio of log_2_-fold (vs that in MM)** | | | |
| --- | --- | --- | --- | --- | --- | --- | --- | --- | --- | --- | --- |
|  |  |  | **4** | **8** | **12** | **16** |  | **4** | **8** | **12** | **16** |
| Twitching motility protein  /T2SS Protein E | *pilT* | *PccS1_00329* | — | — | — | 2.50 |  | — | — | — | — |
| Prepilin peptidase-dependent  protein D | *ppdD* | *PccS1_00456* | — | — | — | — |  | — | — | — | — |
| T2SS protein E | *hofB* | *PccS1_00457* | — | — | — | — |  | — | — | — | — |
| Transport protein | *gspF/pilC/hofC* | *PccS1_00458* | — | — | — | — |  |  | — | — | — |
| Putative DNA transport protein | *hofQ/pilQ* | *PccS1_00841* | 2.22 | — | — | — |  | — | — | — | — |
| rcpA pilus assembly protein | *rcpA/cpaC* | *PccS1_01977* | 3.65 | 2.18 | 4.62 | 4.20 |  | — | -3.35 | — |  |
| tadA pilus assembly protein | *tadA/cpaF* | *PccS1_01980* | 5.00 | 3.73 | 5.89 | 5.55 |  |  | -2.79 |  |  |
| Tight adherence protein B | *tadB* | *PccS1_01981* | 5.91 | 5.22 | 7.15 | 6.89 |  | -2.21 | -2.82 |  | — |
| Tight adherence protein C | *tadC* | *PccS1_01982* | 5.10 | 3.33 | 4.74 | 5.73 |  | — | -3.41 | -2.07 | — |
| P-type DNA transfer ATPase | *virB11* | *PccS1_02801* | — | — | — | — |  | — | — | — | — |
| Necrosis inducing protein | *nip* | *PccS1_0455* | — | 2.88 | 4.21 | 4.05 |  | — | 4.49 | 5.74 | 5.51 |
| T2SS protein N | *outN/gspN* | *PccS1_04168* | 2.30 | — | 2.21 | 2.49 |  | — | — | — | — |
| T2SS protein M | *outM/gspM* | *PccS1_04169* | — | — | 2.47 | — |  | — | — | — | — |
| T2SS protein L | *outL/gspL* | *PccS1_04170* | 2.42 | — | 2.84 | 2.60 |  | — | — | — | — |
| T2SS protein K | *outK/gspK* | *PccS1_04171* | — | — | — | 2.20 |  | — | — | — | — |
| T2SS protein J | *outJ/gspJ* | *PccS1_04172* | — | — | — | — |  | — | — | — | — |
| T2SS protein I | *outI/gspI* | *PccS1_04173* | — | — | — | 2.58 |  | — | — | — | — |
| T2SS protein H | *outH/gspH* | *PccS1_04174* | — | — | — | 2.87 |  | — | — | — | — |
| T2SS protein G | *outG/gspG* | *PccS1_04175* | — | — | — | — |  | — | — | — | — |
| T2SS protein F | *outF/gspF* | *PccS1_04176* | — | — | — | — |  | — | — | — | — |
| T2SS protein E | *outE/gspE* | *PccS1_04177* | — | — | — | — |  | — | — | — | — |
| T2SS protein D | *outD/gspD* | *PccS1_04178* | — | — | — | — |  | — | — | — | — |
| T2SS protein C | *outC/gspC* | *PccS1_04179* | — | — | — | — |  | — | — | — | — |
| T2SS Protein B | *outB/gspC* | *PccS1_04182* | — | — | — | — |  | — | — | — | — |
| T2SS Protein S | *outS/gspS* | *PccS1_04183* | — | — | — | — |  | — | — | — | — |
| Chaperone lipoprotein OutS-like |  | *PccS1_04418* | — | — | — | — |  | — | — | — | — |
