# Supplementary material for: Transcriptome of Pectobacterium carotovorum subsp. carotovorum PccS1 infected in calla plants in vivo highlights a spatiotemporal expression pattern of genes related to virulence, adaptation, and host response
Source: Mol Plant Pathol. 2020 Apr 8;21(6):871–91. doi: 10.1111/mpp.12936 (PMC7214478; doi:10.1111/mpp.12936)
Supplement: Supplementary file 10 — TABLE S6 The sequences of oligonucleotides for molecular modification used in this study [file MPP-21-871-s010.docx]

**Table S6** The sequences of oligonucleotides for molecular modification in this study

| Primer | Sequence (5'-3') with the sites of restriction enzyme underlined | Restriction enzyme |
| --- | --- | --- |
| *00112*-1F | CCCGGTACCACTCAGACCACAACTCAG | *Kpn*I |
| *00112*-1R | CCCGAATTCCAGCCCTTCATCCAGCCT | *Eco*RI |
| *00112*-2F | CCCTCTAGACCAGAAGATCCGACGAAG | *Xba*I |
| *00112*-2R | CCCAAGCTTCAAGTCATCAAGAATAGGC | *Hind*III |
| *00113*-1F | CCCGGTACCCTGGTCGGATAATCGTGG | *Kpn*I |
| *00113*-1R | CCCGAATTCGCCCTGCGTTTTACCTTCG | *Eco*RI |
| *00113*-2F | CCCTCTAGAACGGTTGCCGGTACTTCTG | *Xba*I |
| *00113*-2R | CCCAAGCTTCTCCTTCTTCGCCGTTGC | *Hind*III |
| *00116-*1F | CCCGGTACCTTACTCGCCGAAGAAGGG | *Kpn*I |
| *00116*-1R | CCCGAATTCCCAGACCAGCGTGAACACC | *Eco*RI |
| *00116*-2F | CCCTCTAGACTGGAGAACGGTGCGATGAC | *Xba*I |
| *00116*-2R | CCCAAGCTTGCTGATGCGGGCAATAAA | *Hind*III |
| *00120**-*1F | CCCGGTACCCGACCGTCTTCCACCTGA | *Kpn*I |
| *00120*-1R | CCCGAATTCGATTTCCGCACCCTGATG | *Eco*RI |
| *00120*-2F | CCCTCTAGAAACCGTAGCGAAAATGGG | *Xba*I |
| *00120*-2R | CCCAAGCTTGCATCTGCCGAAAATCACG | *Hind*III |
| *00122-*1F | CCCGGTACCAAATACCTGCCGCCTGAA | *Kpn*I |
| *00122*-1R | CCCGAATTCTGACCTTACCGACCTCCA | *Eco*RI |
| *00122*-2F | CCCTCTAGAACGTTGGCGTTCCACATTG | *Xba*I |
| *00122*-2R | CCCAAGCTTACCCAGGCAGAGGCAGAG | *Hind*III |
| *00132-*1F | CGGGGTACCTGTAAGATAGGCTACGTGAT | *Kpn*I |
| *00132*-1R | CGCGGATCC GATGAGTCCTTGTTTGATG | *Bam*HI |
| *00132*-2F | GCTCTAGA AATGGGAGCATGTGGTTG | *Xba*I |
| *00132*-2R | CCCAAGCTT AGGCGTAAATTGTGAGTGTC | *Hind*III |
| *01155**-*1F | CGGAATTCGTTGAAGGTAAGCAGGAGC | *Eco*RI |
| *01155*-1R | GCTCTAGAAAGGCGTAGTCGGTGATG | *Xba*I |
| *01155*-2F | CGGGGTACCCCTTCCACGCCACCAATC | *Kpn*I |
| *01155*-2R | CCCAAGCTTACCGTAAAAGCGGGTTCG | *Hind*III |
| *03544-*1F | CGCGGATCCGAACTACAAAGCACTGTCTATC | *Bam*HI |
| *03544*-1R | CGGAATTCAAGCATAATGTTTCCTCAA | *Eco*RI |
| *03544*-2F | GCTCTAGAGAAGGCTGTGGGTATGAT | *Xba*I |
| *03544*-2R | CCCAAGCTTACTCTGCTGTCACGGCTA | *Hind*III |
| *03554-*1F | CGCGGATCCAACGAAATCAAAACAAGTC | *Bam*HI |
| *03554*-1R | CGGAATTCGAGAAAGTCCTAAAGCCAT | *Eco*RI |
| *03554*-2F | GCTCTAGATCTGCGTCCAAATCACTG | *Xba*I |
| *03554*-2R | CCCAAGCTTGTACACTTTTCGCCACCA | *Hind*III |
| *03561-*1F | CGCGGATCCCGCTGACACCCTCTATTT | *Bam*HI |
| *03561*-1R | CGGAATTCTTCATGGCTGCTCCTTAT | *Eco*RI |
| *03561*-2F | GCTCTAGACCCTGTTACAAGACGAAT | *Xba*I |
| *03561*-2R | CCCAAGCTTCAACTGCCTACCATCACC | *Hind*III |
| *03562-*1F | CGCGGATCCGACGCAGAACGAAACCTT | *Bam*HI |
| *03562*-1R | CGGAATTCGCACGAGATCGGCATCAT | *Eco*RI |
| *03562*-2F | GCTCTAGAGATAACGGTGAACTGGTAA | *Xba*I |
| *03562*-2R | CCCAAGCTTATCAGTCCGAAAACAATG | *Hind*III |
| *03563-*1F | CGCGGATCCGTGAAAGTGGCGGCGTAT | *Bam*HI |
| *03563*-1R | GCTCTAGACATCTGGCTGTCGTCCTG | *Xba*I |
| *03563*-2F | CGGGGTACCTGATGTGCTGACCGATTC | *Kpn*I |
| *03563*-2R | CCCAAGCTTGTTTCTCCACTGCCTTCT | *Hind*III |
| *00073-*1F | CGGAATTCCTGTTCGGTGAGAAGTAT | *EcoRI* |
| *00073-*1R | CGGGGTACCGAGGGTTTCGCCAACTCG | *Kpn*I |
| *00073-*2F | GCTCTAGAATGCGTCTCGTGTTCGCGG | *Xba*I |
| *00073-*2R | CCCAAGCTTTTACTCGGCTTCGCCTTG | *Hind*III |
| *00230-*1F | CGGGGTACCCCAAATCCGCACCGATCA | *Kpn*I |
| *00230-*1R | GCTCTAGACATAAGTGGCTCCTACCC | *Xba*I |
| *00230-*2F | GCTCTAGATGAGCATTGAAGAACTGT | *EcoRI* |
| *00230-*2R | CCCAAGCTTTCAGGTAGCCAGCCCACTG | *Hind*III |
| *00262-*1F | CGGAATTCAGCAGGCTCATCAACAGG | *Eco*RI |
| *00262-*1R | CGGGATCCTGCCGATAGACTTCCACAA | *Bam*HI |
| *00262-*2F | GCTCTAGACTGTGGCGATTACAGCACG | *Xba*I |
| *00262-*2R | GGGGTACCCGGTCAGGAAGCCCATCA | *Kpn*I |
| *01441-*1F | GCTCTAGAGACCGAGCCGTCTTCATT | *Xba*I |
| *01441-*1R | GGGGTACCCAGCGTTCGCAGGTGTTT | *Kpn*I |
| *01441-*2F | CGGGATCCGAGCACTTACCGTTTGTGAA | *Bam*HI |
| *01441-*2R | CCCAAGCTTCTATAAGCGCCTGATGGAG | *Hin*dIII |
| *01523-*1F | GCTCTAGAACCCGCATAAAGCCAGAG | *Xba*I |
| *01523-*1R | GGGGTACCAACGGTCAGTACGTCAGAAT | *Kpn*I |
| *01523-*2F | CGGGATCCAATACGGCATGAACTGATA | *Bam*HI |
| *01523-*2R | CCCAAGCTTTCAAATGACATCGCTTCT | *Hin*dIII |
| *02702-*1F | GCTCTAGACAATGCCAAACGCCTCAT | *Xba*I |
| *02702-*1R | GGGGTACCTATTCGCCACCTCGGTCA | *Kpn*I |
| *02702-*2F | CGGGATCCAACGATGAAGCGGTGATT | *Bam*HI |
| *02702-*2R | CCCAAGCTTAACGCGATAAGGATTTGC | *Hin*dIII |
| *03188-*1F | CCCGGTACCTGAACGCTTTCGTGTCTT | *Kpn*I |
| *03188-*1R | CCCGAATTCTTTTCTTTCCGATTTGCT | *Eco*RI |
| *03188-*2F | CCCTCTAGACTGGTTGAAATGGCACAGC | *Xba*I |
| *03188-*2R | CCCAAGCTTAAAGATGAAGCGGGTAGATG | *Hin*dIII |
| *03516-*1F | GCTCTAGATGCGTGATGACTTCCGTGAA | *Xba*I |
| *03516-*1R | GGGGTACCGCGATCTTTGCTGCTCTTT | *Kpn*I |
| *03516-*2F | CGGGATCCGGAAGTGGCTCCACCTGA | *BamHI* |
| *03516-*2R | CCCAAGCTTCCCGCCGTCTTCACTCAT | *Hind*III |
| *03556-*1F | GCTCTAGATATTCTTTCGCTTTCTTGTG | *Xba*I |
| *03556-*1R | GGGGTACCGGTTTCACCTTCCAGCAC | *Kpn*I |
| *03556-*2F | CGGGATCCACTGAATGTGACGGTGCG | *BamHI* |
| *03556-*2R | CCCAAGCTTCGATTATCTGAGCCATTTACC | *Hind*III |
| *03386-*1F | CGGGGTACCAATCACGGTGCCAGAACG | *Kpn*I |
| *03386-*1R | CGGAATTCACTGGCAAACCGCCTTAC | *Eco*RI |
| *03386-*2F | GCTCTAGAGCCGAACACCCAAATCCT | *Xba*I |
| *03386-*2R | CCCAAGCTTCGTGCCGTAGTCAAAACC | *Hind*III |
| *03912-*1F | GCTCTAGATCCAGGCATTTGCTTCTT | *Xba*I |
| *03912-*1R | GGGGTACCTGCATTCCGACTTTAGGC | *Kpn*I |
| *03912-*2F | CGGGATCCAGCTCACTCGCACGTAATCC | *BamHI* |
| *03912-*2R | CCCAAGCTTTCCAGAACATCCGCACCA | *Hind*III |
| *03557-*1F | GCTCTAGACCACCTGTCTGCTACGAAC | *Xba*I |
| *03557-*1R | GGGGTACCATCTGCTTGAGCCCTTCA | *Kpn*I |
| *03557-*2F | CGGGATCCGTAAGAAGCTGATTCCCTAG | *BamHI* |
| *03557-*2R | CCCAAGCTTCGAATGATGACCGAAAGT | *Hind*III |
| *04012-*1F | CGGAATTCCCCGTGTTGAGGGTAAAG | *Eco*RI |
| *04012-*1R | CGGGATCCAGCCGTCTAAGATTCATGTCTG | *BamHI* |
| *04012-*2F | GCTCTAGACAATCAATGAGTGCGTAA | *Xba*I |
| *04012-*2R | GGGGTACC CAGCACCTTCTATCTTTCTA | *Kpn*I |
| *01907-*1F | CGGGGTACCCACTGAGTTCGCAGGGTA | *Kpn*I |
| *01907-*1R | CGGAATTC TGTCTTCCCACCGCACCA | *Eco*RI |
| *01907-*2F | GCTCTAGACGAACACGGCACGCTAAC | *Xba*I |
| *01907-*2R | CCCAAGCTT CCAGATGTCACCAGCGTC | *Hind*III |
| *01908-*1F | GGGGTACCGCGTTATTGGTTTTCGTAGT | *Kpn*I |
| *01908-*1R | GCTCTAGATATCCGCACGCTTAACCGCC | *Xba*I |
| *01908-*2F | CGGAATTCTCAAGGCGTATGCGGAAGCG | *Eco*RI |
| *01908-*2R | CCCAAGCTTCCTGCTGAAAGATAAGTGTT | *Hind*III |
| *03264-*1F | GCTCTAGAGACGCCTCACCACAAACT | *Xba*I |
| *03264-*1R | GGGGTACCATACCCTGACCCAAACCC | *Kpn*I |
| *03264-*2F | CGGGATCCCAGTATTCCTGGCTTCCA | *BamHI* |
| *03264-*2R | CCCAAGCTTATAGCGTCGGCTTAGTTT | *Hind*III |
| *03817-*1F | CGGGGTACCGTTCCTGCTCAGCCTTCG | *Kpn*I |
| *03817-*1R | CGGAATTCATAACATTTTGAGCGAGA | *Eco*RI |
| *03817-*2F | GCTCTAGAGTGCCTGTCTGCTCCTGT | *Xba*I |
| *03817-*2R | CCCAAGCTTCGTCATCGGGCGTCAAAT | *Hind*III |
| *04053-*1F | CGGGGTACCTTCAATCCCGAGCAAGCC | *Kpn*I |
| *04053-*1R | CGGGATCCCGCGTTACCACCACTACCGT | *BamHI* |
| *04053-*2F | GCTCTAGAGTTGTACGCATCGTCGGC | *Xba*I |
| *04053-*2R | CGGGGTACCTTCAATCCCGAGCAAGCC | *Hind*III |
| *04054-*1F | CGGGGTACCCGGTTCATCCATCAGCAA | *Kpn*I |
| *04054-*1R | CGGGATCCCGTTGAGCAGGGACTGGTGT | *BamHI* |
| *04054-*2F | GCTCTAGA GGTTTCTGGAGCGTTATC | *Xba*I |
| *04054-*2R | CCCAAGCTTTCCGATGGTATGTGGTTC | *Hind*III |
| *04055-*1F | CGGGGTACCAATCAGGAACCCCGTAGG | *Kpn*I |
| *04055-*1R | CGGGATCCCGGGAAATGTGCCGTTACCC | *BamHI* |
| *04055-*2F | GCTCTAGATCAACCCTGGCAGCATCA | *Xba*I |
| *04055-*2R | CCCAAGCTTAGAAACCGCCCCTGAGAA | *Hind*III |
| *Km-*F | CCCGAATTCGAAGCTCCCTCGTGC | *Eco*RI |
| *Km-*R | CCCTCTAGACAGGTGGCACTTTTCG | *Xba*I |
| *Km-*F1 | GCTCTAGAGAAGCTCCCTCGTGC | *Xba*I |
| *Km-*R1 | CCCGAATTCGAAGCTCCCTCGTGC | *Eco*RI |
| *Km-*F2 | CGGGGTACCCAGGTGGCACTTTTCG | *Kpn*I |
| *Km*-R2 | CGCGGATCCGAAGCTCCCTCGTGC | *Bam*HI |
| *03557-*F | CGGGATCCATCAAAGACAACGGCAAAGG | *Bam*HI |
| *03557-*R | GCTCTAGACTAGGGAATCAGCTTCTTAC | *Xba*I |
| *Pcc-*F | TGTAGCGGCCTACCTGTCTG |  |
| *Pcc-*R | TCAATCCCAGTTTCTGACCG |  |
| *Gm-*F | GTTAGGTGGCGGTACTTGGGTCG |  |
| *Gm-*R | ATGTTACGCAGCAGCAACGATGT |  |
