# Supplementary material for: Transcriptome of Pectobacterium carotovorum subsp. carotovorum PccS1 infected in calla plants in vivo highlights a spatiotemporal expression pattern of genes related to virulence, adaptation, and host response
Source: Mol Plant Pathol. 2020 Apr 8;21(6):871–91. doi: 10.1111/mpp.12936 (PMC7214478; doi:10.1111/mpp.12936)
Supplement: Supplementary file 11 — TABLE S7 Bacterial strains and plasmids used in this study [file MPP-21-871-s011.docx]

**Table S7** Bacterial strains and plasmids used in this study

| Strains /plasmids | Relevant characteristics | Source or reference |
| --- | --- | --- |
| **Strains**  *Pectobacterium carotovorum* subsp*. carotovorum* | |  |
| PccS1 | Wild type strain, Rif^r^ | ([Gu *et al.*, 2009](#_ENREF_1)) |
| Δ*00112* | Rif^r^, Gm^r^, *00112* gene knockout mutant of strain PccS1 | This study |
| Δ*00113* | Rif^r^, Gm^r^, *00113* gene knockout mutant of strain PccS1 | This study |
| Δ*00116* | Rif^r^, Gm^r^, *00116* gene knockout mutant of strain PccS1 | This study |
| Δ*00120* | Rif^r^, Gm^r^, *00120* gene knockout mutant of strain PccS1 | This study |
| Δ*00122* | Rif^r^, Gm^r^, *00122* gene knockout mutant of strain PccS1 | This study |
| Δ*00132* | Rif^r^, Gm^r^, *00132* gene knockout mutant of strain PccS1 | This study |
| Δ*01155* | Rif^r^, Gm^r^, *01155* gene knockout mutant of strain PccS1 | This study |
| Δ*03544* | Rif^r^, Gm^r^, *03544* gene knockout mutant of strain PccS1 | This study |
| Δ*03554* | Rif^r^, Gm^r^, *03554* gene knockout mutant of strain PccS1 | This study |
| Δ*03561* | Rif^r^, Gm^r^, *03561* gene knockout mutant of strain PccS1 | This study |
| Δ*03562* | Rif^r^, Gm^r^, *03562* gene knockout mutant of strain PccS1 | This study |
| Δ*03563* | Rif^r^, Gm^r^, *03563* gene knockout mutant of strain PccS1 | This study |
| Δ*00073* | Rif^r^, Gm^r^, *00073* gene knockout mutant of strain PccS1 | This study |
| Δ*00230* | Rif^r^, Gm^r^, *00230* gene knockout mutant of strain PccS1 | This study |
| Δ*00262* | Rif^r^, Gm^r^, *00262* gene knockout mutant of strain PccS1 | This study |
| Δ*01441* | Rif^r^, Gm^r^, *01441* gene knockout mutant of strain PccS1 | This study |
| Δ*01523* | Rif^r^, Gm^r^, *01523* gene knockout mutant of strain PccS1 | This study |
| Δ*02702* | Rif^r^, Gm^r^, *02702* gene knockout mutant of strain PccS1 | This study |
| Δ*03188* | Rif^r^, Gm^r^, *03188* gene knockout mutant of strain PccS1 | This study |
| Δ*03516* | Rif^r^, Gm^r^, *03516* gene knockout mutant of strain PccS1 | This study |
| Δ*03556* | Rif^r^, Gm^r^, *03556* gene knockout mutant of strain PccS1 | This study |
| Δ*03836* | Rif^r^, Gm^r^, *03836* gene knockout mutant of strain PccS1 | This study |
| Δ*03912* | Rif^r^, Gm^r^, *03912* gene knockout mutant of strain PccS1 | This study |
| Δ*03557* | Rif^r^, Gm^r^, *03557* gene knockout mutant of strain PccS1 | This study |
| Δ*04012* | Rif^r^, Gm^r^, *04012* gene knockout mutant of strain PccS1 | This study |
| Δ*01907* | Rif^r^, Gm^r^, *01907* gene knockout mutant of strain PccS1 | This study |
| Δ*01908* | Rif^r^, Gm^r^, *01908* gene knockout mutant of strain PccS1 | This study |
| Δ*03264* | Rif^r^, Gm^r^, *03264* gene knockout mutant of strain PccS1 | This study |
| Δ*03817* | Rif^r^, Gm^r^, *03817* gene knockout mutant of strain PccS1 | This study |
| Δ*04053* | Rif^r^, Gm^r^, *04053* gene knockout mutant of strain PccS1 | This study |
| Δ*04054* | Rif^r^, Gm^r^, *04054* gene knockout mutant of strain PccS1 | This study |
| Δ*04055* | Rif^r^, Gm^r^, *04055* gene knockout mutant of strain PccS1 | This study |
| Δ*0*3557(*03557*) | Rif^r^, Gm^r^, Km^r^, Δ*03557* complemented with pBBR-*03557* | This study |
| Δ*03557*(pBBR) | Rif^r^, Gm^r^, Km^r^, Δ*03557* complemented with pBBR1MCS5 | This study |
| *Escherichia coli* | |  |
| DH5α | Φ80 lacZΔM15, (lacZYA-argF) U169. recA1,endA1.thi-1 | TaKaRa |
| S17-1λpir | Sp^r^, λ-pir Lysogen of S17-1 | TaKaRa |
| **Plasmids** |  |  |
| pEX18Gm | Gm^r^, allelic exchange suicide vector, *sacB* oriT (RP4) *LacZ,* | ([Hoang *et al.*, 1998](#_ENREF_2)) |
| pET30a | Km^r^, Template plasmid for kanamycin cassette | TaKaRa |
| pBBR1MCS5 | Gm^r^, broad-host-range cosmid vector | ([Kovach *et al.*, 1995](#_ENREF_3)) |
| pBBR-*03557* | Gm^r^, pBBR1MCS5 derivative with PCR fragment containing *03557* | This study |

**Gu, C., Fan, J., Yang, X., Hu, B. and Liu, F.** (2009) Identification of the pathogen and quorum quenching study on bacterial soft rot of colored calla lily. *J of Nanjing Agric Univ*. **32**, 71-77.

**Hoang, T.T., Karkhoff-Schweizer, R.R., Kutchma, A.J. and Schweizer, H.P.** (1998) A broad-host-range Flp-FRT recombination system for site-specific excision of chromosomally-located DNA sequences: application for isolation of unmarked *Pseudomonas aeruginosa* mutants. *Gene*. **212**, 77-86.

**Kovach, M.E., Elzer, P.H., Hill, D.S., Robertson, G.T., Farris, M.A., Roop, R.M., 2nd and Peterson, K.M.** (1995) Four new derivatives of the broad-host-range cloning vector pBBR1MCS, carrying different antibiotic-resistance cassettes. *Gene*. **166**, 175-6.
