# Supplementary material for: Transcriptome of Pectobacterium carotovorum subsp. carotovorum PccS1 infected in calla plants in vivo highlights a spatiotemporal expression pattern of genes related to virulence, adaptation, and host response
Source: Mol Plant Pathol. 2020 Apr 8;21(6):871–91. doi: 10.1111/mpp.12936 (PMC7214478; doi:10.1111/mpp.12936)
Supplement: Supplementary file 12 — TABLE S8 The sequences of RT‐qPCR primers used in this study [file MPP-21-871-s012.docx]

**Table S8** The sequences of RT-qPCR primers used in this study

| Primer | Sequence (5'-3') |
| --- | --- |
| *recA*-RT-F | TCTGGGTGCCGGTGGTT |
| *recA*-RT-R | TCACAGATTTCCAGCGCTTGC |
| *hlyD-*RT-F | GAGGTAGCGGCGGTATTC |
| *hlyD-*RT-R | GGCGTAAGCGCAGATGTT |
| *tadB-*RT-F | GAACTACAAAGCACTGTCTATC |
| *tadB-*RT-R | CACTCATCCACGCTAACC |
| *tadC-*RT-F | TGGTGGTCTATCGCTGGTT |
| *tadC-*RT-F | ATCCGTCAGAATCGCAGA |
| *hrpA*-RT-F | ATGGCTTTAGGACTTTCTC |
| *hrpA*-RT-R | GTTCAGTGATTTGGACGC |
| *hrpB*-RT-F | ACGGCAGAAAGCGGTAAT |
| *hrpB*-RT-R | TTCGGGTCGCATCCAACA |
| *hrcC*-RT-F | TGGTGGTCTATCGCTGGTT |
| *hrcC*-RT-R | ATCCGTCAGAATCGCAGA |
| *hrpD*-RT-F | CGGTATTACGCCTGATGG |
| *hrpD*-RT-R | CGCCGAGATAAGGTGATGAT |
| *hrpE*-RT-F | CGTTTGTGACATTACCCG |
| *hrpE*-RT-R | CGCTTCTGCCTGTTCTACT |
| *hrcJ*-RT-F | GAGAAACGGGTGGACAAA |
| *hrcJ*-RT-R | AGACCTCGCCCAGATTAG |
| *dotU*-RT-F | ATCGCGACATTCTGGAGTTT |
| *dotU* -RT-R | CGTAGCGTGTCATGCAACTT |
| *pilQ-*RT-F | CAGAAATTACCGCTGCACAA |
| *pilQ-*RT-R | TGCAGAGAGCAATGTTCCAC |
| *vgrG*-RT-F | ACTGCGTTTTGAGGATGAGG |
| *vgrG*-RT-R | CCGATGGATGTTGTTGATGA |
| *hcp*-RT-F | CGTCGGTTGAAGGTAAGCA |
| *hcp*-RT-R | TGGGTGTAGTCCAGTTTTGATG |
| *clpB*-RT-F | CCCGCTATACCCAGAACGTA |
| *clpB*-RT-R | TTACGACGGCGAGAGAGAAT |
| *impH*-RT-F | GGTCGGCAACCTCAGTTTTA |
| *impH*-RT-R | CAACTGATCGCGAAGAATGA |
| *impG*-RT-F | GTGGAAGGCTGGGTTGAA |
| *impG*-RT-R | CCCGAATGCGGTAATAAA |
| *impC*-RT-F | ACCAAAGAAGGCAAAGAAGC |
| *impC*-RT-R | GCCAATCTGTTCACGCTGTA |
| *impB*-RT-F | TGAAGAAGAAAGCCAGGATGA |
| *impB*-RT-R | GTTGGGCGATACGGTCAG |
| *vgrG2*-RT-F | CAGCCTGCAAGTGAAAAACA |
| *vgrG2*-RT-R | GTGACGGTAGTGCCCTTGAT |
| *hcp4*-RT-F | TCGGCAACATCTATGTGGAA |
| *hcp4*-RT-R | GGTGAATTTGAACGGCTTGT |
| *vgrG3*-RT-F | TGGTGTGGTACGAAGGTGAA |
| *vgrG3*-RT-R | GGACGGACTTCTGCCTGATA |
| *celV*-RT-F | CAATCCTTCCCTTGCGAATA |
| *celV*-RT-R | AATGTGCCAGTCGATGATGA |
| *prtW*-RT-F | AGGTTAGCCCTACCCAAAA |
| *prtW*-RT-R | ACCTTCGCCAGCATTGTA |
| *pel1*-RT-F | CAACGGTTCTTCCGCTAA |
| *pel1*-RT-R | GATTCAAAGGTCGTGTCG |
| *Pel3*-RT-F | AAGATCTGAGCCGCTCTCTG |
| *Pel3*-RT-R | AGATTGCCGATCAATTCCAG |
| *fliP*-RT-F | TGATGTTTGCGCTCTTTCTG |
| *fliP*-RT-R | CATATTGGGCGGTACTTGCT |
| *vcpD*-RT-F | CCCGTTATTCAGTTGGCTGT |
| *vcpD*-RT-R | ACTTCCACCAGCGGATACTG |
| *kdgM*-RT-F | TAATGAAACCGTCAGCAACG |
| *kdgM*-RT-R | CCACGTAGATAAGGGCGGTA |
| *rplY*-RT-F | GCGTACGAAGTCGATGTGAG |
| *rplY*-RT-R | AAATTCCCAGCCATCGTTTA |
| *glgB*-RT-F | GCAATGGACGAAAAATCGTT |
| *glgB*-RT-R | ATCACCCAGGTTTCTTCACG |
| *03544-*RT-F | GGTTTGTTGGGCGGAGGA |
| *03544-*RT-R | CGACGCTGGCTGCTTGTT |
| *03554-*RT-F | ATGGCTTTAGGACTTTCTC |
| *03554-*RT-R | GTTCAGTGATTTGGACGC |
| *03561-*RT-F | GGTCGTTGGTGCGGTCAT |
| *03561-*RT-R | AGCGGCTTGGGCAGATAC |
| *03562-*RT-F | CTACACCCACGCCACGCTT |
| *03562-*RT-R | TACGCCGCCACTTTCACG |
| *03563-*RT-F | CTGCTGATTATTGATTCCC |
| *03563-*RT-R | GTGATGGCACCGTCTTCC |
